# SOX9 suppresses colon cancer via inhibiting epithelial-mesenchymal transition and SOX2 induction

**DOI:** 10.1172/JCI184115

**Published:** 2025-04-03

**Authors:** Ying Feng, Ningxin Zhu, Karan Bedi, Jinju Li, Chamila Perera, Maranne Green, Naziheh Assarzadegan, Yali Zhai, Qingzhi Liu, Veerabhadran Baladandayuthapani, Jason R. Spence, Kathleen R. Cho, Eric R. Fearon

**Affiliations:** 1 Department of Internal Medicine, University of Michigan Medical School, Ann Arbor, Michigan, USA.; 2 Department of Biostatistics, University of Michigan School of Public Health, Ann Arbor, Michigan, USA.; 3 Department of Pathology,; 4 Department of Computational Medicine and Bioinformatics,; 5 Rogel Cancer Center,; 6 Department of Cell and Developmental Biology, and; 7 Department of Human Genetics, University of Michigan Medical School, Ann Arbor, Michigan, USA.

**Keywords:** Gastroenterology, Genetics, Oncology, Colorectal cancer, Mouse models, Tumor suppressors

## Abstract

The Wnt/β-catenin pathway regulates expression of the *SOX9* gene, which encodes sex-determining region Y–box (SOX) transcription factor 9, a differentiation factor and potential β-catenin regulator. Because APC tumor suppressor defects in approximately 80% of colorectal cancers (CRCs) activate the Wnt/β-catenin pathway, we studied SOX9 inactivation in CRC biology. Compared with effects of *Apc* inactivation in mouse colon tumors, combined *Apc* and *Sox9* inactivation instigated more invasive tumors with epithelial-mesenchymal transition (EMT) and SOX2 stem cell factor upregulation. In an independent mouse CRC model with combined *Apc*, *Kras*, and *Trp53* defects, *Sox9* inactivation promoted SOX2 induction and distant metastases. About 20% of 171 human CRCs showed loss of SOX9 protein expression, which correlated with higher tumor grade. In an independent group of 376 patients with CRC, low *SOX9* gene expression was linked to poor survival, earlier age at diagnosis, and increased lymph node involvement. *SOX9* expression reductions in human CRC were linked to promoter methylation. EMT pathway gene expression changes were prominent in human CRCs with low *SOX9* expression and in a mouse cancer model with high SOX2 expression. Our results indicate SOX9 has tumor suppressor function in CRC; its loss may promote progression, invasion, and poor prognosis by enhancing EMT and stem cell phenotypes.

## Abstract 2

This study shows loss of SOX9 function leads to increased invasion and metastasis in colorectal cancer via reduced epithelial differentiation and enhanced cancer stemness.

## Introduction

Colorectal cancer (CRC) is the third most frequently diagnosed cancer and second leading cause of cancer-related mortality globally (1). Key recurrent molecular alterations contributing to CRC development and progression have been identified, but further progress is needed to clarify the functional contributions of the defects in cancer phenotypes and patient outcomes. About 80% of sporadic CRCs harbor inactivating mutations in the adenomatous polyposis coli (*APC*) tumor suppressor gene, and *APC* mutations are believed to be early and potentially rate-limiting events in CRC development (2). The APC protein functions in the β-catenin destruction complex, facilitating efficient phosphorylation and ubiquitination of β-catenin, leading to its subsequent degradation by the proteasome. APC inactivation results in β-catenin accumulation, with β-catenin binding to T cell factor (TCF) transcription factors and altered TCF-regulated gene expression (3–5). The *SOX9* gene, which encodes a sex-determining region Y–box (SOX) transcription factor protein with a highly conserved high mobility group (HMG) box DNA-binding domain and a C-terminal transactivation domain, is activated by β-catenin/TCF signaling (6).

The *SOX9* gene is mutated in approximately 5%–10% of human CRC primary tumors (7–9) and approximately 20% of CRC cell lines (10), and most mutations are frameshift or nonsense mutations in one *SOX9* allele. The loss-of-function mutations suggest a presumptive tumor suppressor role for SOX9 in CRC. But most CRCs and CRC cell lines with *SOX9* mutations retain SOX9 protein expression, as they carry only one mutant *SOX9* allele. Ectopic overexpression of SOX9 in human CRC cells inhibits Wnt/β-catenin signaling activity, leading to downregulation of Wnt target genes (9, 11). There are uncertainties about whether these suppressive effects of ectopic SOX9 on Wnt signaling arise uniquely from its ability to antagonize β-catenin activity and stability, or whether SOX9’s regulatory effects on other genes, such as CEACAM1 (12) and Wnt pathway inhibitors, like ICAT and Groucho-related corepressor (Grg/TLE) (11), may exert an inhibitory effect on β-catenin/TCF activity. Some studies suggest SOX9 can suppress the Wnt/β-catenin signaling pathway by interfering with β-catenin’s binding to TCF4 (13) or chromatin (9). Despite the human CRC tumor data indicating *SOX9* could function as a tumor suppressor gene, most functional studies and data reflect work with SOX9 ectopic overexpression approaches in tissue culture settings. Furthermore, in apparent opposition to the view that *SOX9* may function in CRC as a tumor suppressor gene, some data suggest *SOX9* could have a protumorigenic role in some tissues (14–16). In most human colorectal adenomas and CRCs, SOX9 is highly expressed, but the correlation between SOX9 overexpression and poor prognosis remains contentious (17–19). Two recent studies concluded SOX9 is an oncogenic factor and promotes CRC by activating an aberrant stem cell–like program and interfering with differentiation (17, 20). Some earlier studies also reported an oncogenic role for SOX9, based on the observation that SOX9 inhibition via RNA-inhibitory approaches reduced cell growth and the tumorigenic potential of CRC cells in immunocompromised mice (21, 22).

Given SOX9’s reported functional connections to the Wnt/β-catenin signaling pathway and uncertainties about its potential roles in CRC, we explored how *Sox9* inactivation affects *Apc* mutation–dependent tumorigenesis in the mouse colon as well as the role of SOX9 inactivation in human CRCs. We found that combined inactivation of *Sox9 and Apc* in mouse colon epithelium leads to more advanced, dysplastic, and invasive tumors compared with *Apc* mutation alone. The relevance of our mouse model findings is reflected by marked reduction or loss of SOX9 protein expression in approximately 20% of primary CRCs, and the subset of patients with CRC whose cancers show marked reduction in *SOX9* gene expression have poor prognosis. Collectively, our results indicate SOX9 can function as a tumor suppressor factor in colon tumorigenesis, and SOX9 loss promotes tumor progression in part by augmenting epithelial-mesenchymal transition (EMT) and cancer stemness phenotypes.

## Results

### Biallelic inactivation of both Apc and Sox9 in mouse colon epithelium results in more aggressive tumor features than Apc inactivation alone.

**Figure 1:**
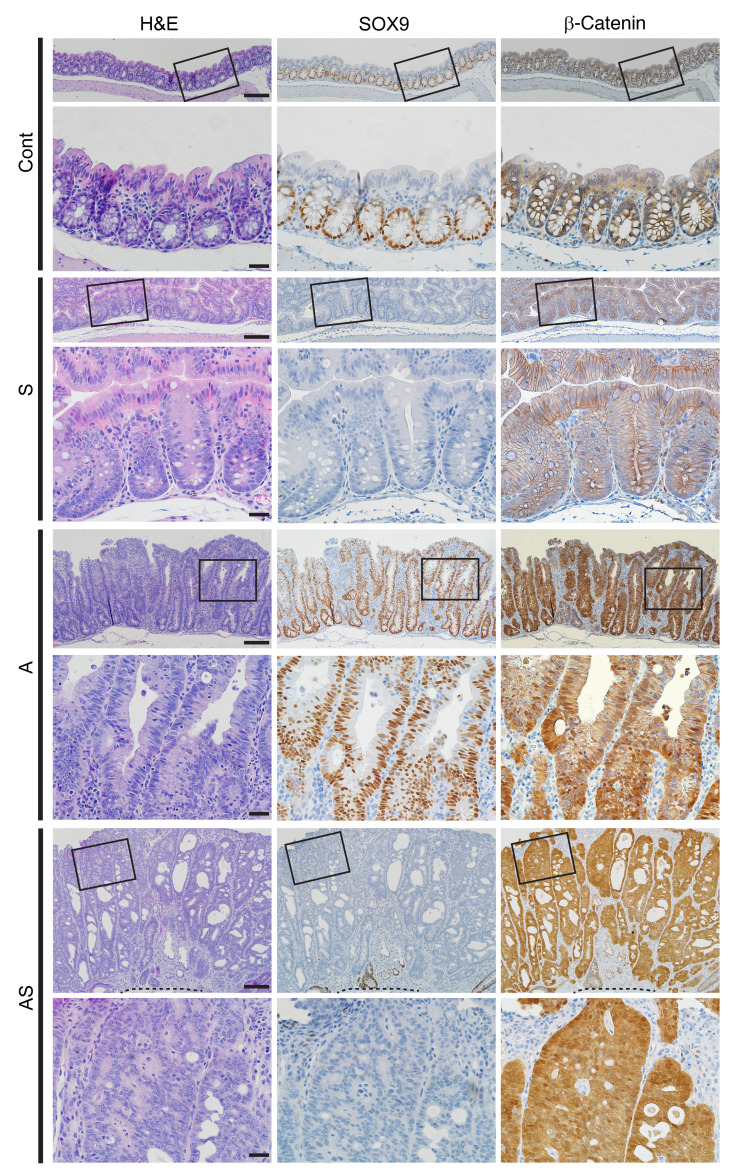
Morphological changes in colon epithelium in mice with inactivation of *Sox*9 (S), *Apc* (A), or both *Apc* and *Sox9* (AS). H&E staining (left) and IHC staining for SOX9 (middle) and β-catenin (right) in proximal colon tissues from S, A, and AS mice, along with a Cre-negative littermate control (Cont) at 35 days after 2 daily doses of TAM to induce Cre-mediated gene targeting. For each mouse, representative images with low magnification (top rows) and corresponding boxed areas at high magnification (directly below) are presented. The dashed lines indicate the muscularis mucosae. Scale bars: 100 μM for low-magnification images; 20 μM for high-magnification images.

**Figure 2:**
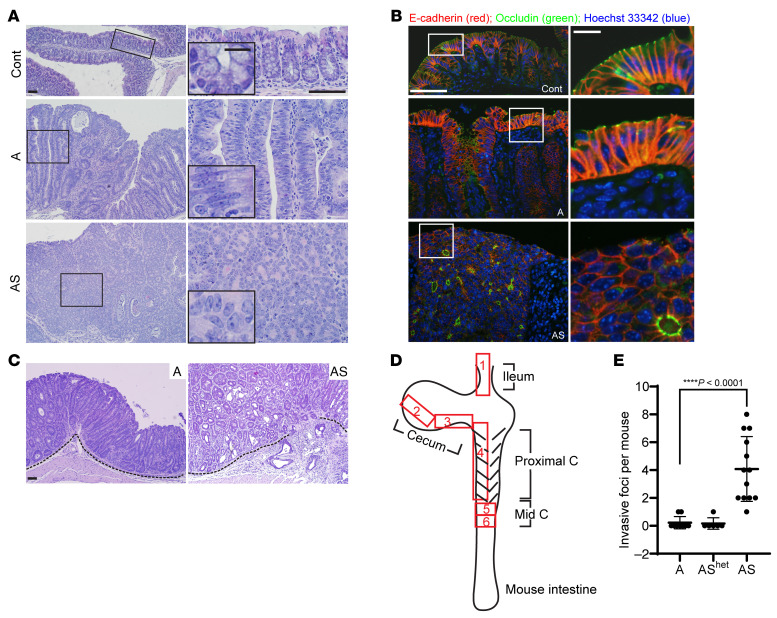
Mouse colon lesions with combined *Apc* and *Sox9* inactivation had enhanced dysplastic changes and invasive features compared with lesions with *Apc* inactivation. (**A**) H&E staining of proximal colon tissues from a control (Cont) mouse and mice with *Apc* inactivation (A) or *Apc* and *Sox9* inactivation (AS), at 35 days after TAM injection for gene inactivation. Representative photomicrographs of low-power magnification are shown in the left panels. The right panels represent high-power magnification of the boxed areas from the left panels, with insets highlighting epithelial features. Scale bars: 50 μm for both low- and high-magnification images; 10 μm for insets. (**B**) Immunofluorescence staining of E-cadherin (red) and occludin (green), counterstained with Hoechst 33342 (blue) to mark nuclei, in colon tissues from the mice described in **A**. Scale bars: 50 μm for low magnification images (left); 10 μm for high magnification images (right). (**C**) Photomicrographs of H&E-stained colon sections show noninvasive polypoid lesions in an A mouse (left) and emerging invasive foci in an AS mouse (right), taken at 35 days and 29 days, respectively, after TAM injection. The dashed lines delineate the muscularis mucosae. Scale bars: 50 μm. (**D**) Diagram of mouse intestinal tract and 6 independent surgical sampling areas (each 10–20 mm long and 5 mm wide) from ileum, cecum, proximal colon, and mid-colon where invasion was assessed. (**E**) Quantification of invasive foci per mouse in colon, cecum, and ileum tissues from the surgical areas described in **D** for A mice (*n* = 9), AS^het^ mice (*n* = 6), and AS mice (*n* = 13) at 28–40 days after TAM injection. Invasive foci were counted in all 6 surgical areas from each mouse (1 tissue section per area). ****P < 0.0001 (Welch’s *t* test: AS mice versus A mice); the data are shown as mean ± SD.

We sought to assess how *Sox9* genetic inactivation might affect *Apc* mutation–dependent mouse colon tumorigenesis. We previously described *CDX2P-CreER^T2^* transgenic mice with a tamoxifen-regulated (TAM-regulated) Cre protein (*CreER^T2^*) under control of human *CDX2* sequences, allowing for TAM-inducible targeting of *loxP*-containing alleles in adult terminal ileum, cecum, colon, and rectal epithelium (23). After TAM dosing of *CDX2P-CreER^T2^* mice with 2 floxed *Sox9* alleles (for Cre-mediated deletion of *Sox9* exons 2 and 3), we found *Sox9* biallelic inactivation (designated as “S”) had no clear effects on proximal colon epithelial gland morphology compared with control epithelium (designated as “Cont”), with only a modest increase in crypt height in S versus Cont epithelium (Figure 1). In the proximal colon of *CDX2P-CreER^T2^* *Apc^fl/fl^* mice, after TAM-induced biallelic *Apc* gene targeting (designated as “A”), we found thickened epithelium and polypoid lesions arising (Figure 1). We also generated compound *CDX2-CreER^T2^* *Apc^fl/fl^* *Sox9^fl/fl^* mutant mice, allowing for TAM-induced concurrent inactivation of *Apc* and *Sox9* (designated as “AS”). Compared with A mice, colon mucosa of AS mice was more thickened, with more crypt fission and high-grade dysplasia (intramucosal carcinoma), reflected by prominent nuclear atypia (increased nuclear-to-cytoplasmic ratio, loss of polarity, and vesicular nuclei with prominent nucleoli) and multifocal cribriform glandular architecture (Figure 1 and Figure 2A).

We used IHC to evaluate SOX9 and β-catenin protein expression in colon tissues at 28–40 days after TAM treatment. Compared to localized SOX9 nuclear expression only in crypt base epithelial cells in Cont mice, increased SOX9 nuclear staining was seen in epithelial cells throughout crypts of A mice (Figure 1). As expected, in AS and S colon epithelium, there was complete loss of SOX9 expression (Figure 1). Also, as expected based on the APC protein’s role in regulating β-catenin levels and localization, IHC analysis of A and AS mouse colon tissues showed strong cytoplasmic and nuclear β-catenin expression in nearly all epithelial cells. In colon epithelium of Cont and S mice, chiefly membrane β-catenin staining was seen (Figure 1).

Expression of the tight junction protein occludin was misaligned and disorganized in AS mice, compared with ordered occludin localization at the apical region of luminal epithelial cells in A mice (Figure 2B). Moreover, we found all 13 AS mice studied at 29–37 days after TAM treatment had at least 1 invasive focus where tumor cells had invaded through the muscularis mucosae, with an average of 4 invasive foci per mouse (Figure 2, C–E). We found rare invasive foci in A mice euthanized at 28–40 days after treatment, with only 2 of 9 A mice each having 1 invasive focus (*P* < 0.0001 compared with AS mice, Figure 2, C–E). Furthermore, although the colon mucosa in A mice euthanized at 70–100 days after TAM treatment was thicker and more dysplastic relative to the 28–40 day post-TAM treatment period, we found no increase in the number of invasive foci per mouse in older A mice compared with those euthanized at 28–40 days (Figure 2E and Supplemental Figure 1; supplemental material available online with this article; https://doi.org/10.1172/JCI184115DS1). The data suggest *Sox9* inactivation promotes tumor progression rather than simply exacerbating phenotypes instigated by *Apc* inactivation. Colon mucosa from mice with inactivation of both *Apc* alleles and heterozygous inactivation of a *Sox9* allele (AS^het^ mice) showed hyperplastic, dysplastic, and invasive features like A mice, both at early and late time points (Figure 2E and Supplemental Figure 1). Our findings support a tumor suppressor role for *Sox9* in *Apc* mutation–dependent tumorigenesis. Combined *Sox9* and *Apc* biallelic inactivation led to lesions with high-grade dysplasia and invasion typical of carcinoma. AS mice had a significantly shortened lifespan relative to A mice (*P* < 0.0001), with a median survival after TAM treatment of 32 days in AS mice versus 67.5 days in A mice (Supplemental Figure 2). The AS^het^ mice had a median survival of 97 days after TAM treatment (*P* = 0.0043; Supplemental Figure 2), suggesting potential differential *Sox9* gene dosage effects on colon epithelial water transport function and resultant mouse dehydration status in AS^het^ versus A mice, given that dehydration was a major factor underlying humane euthanasia endpoints in these mice. The S mice were healthy, lacked any demonstrable colon lesions, and could live more than 16 months after TAM injection (Supplemental Figure 2).

### Altered proliferation and apoptosis in colon epithelium with individual and combined inactivation of Apc and Sox9.

**Figure 3:**
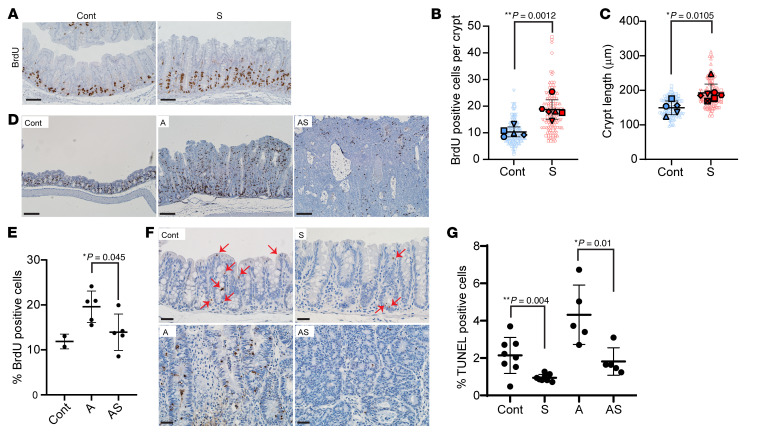
Proliferation and apoptosis changes in mouse colon epithelium with *Apc* (A) and/or *Sox9* inactivation (AS and S). (**A**) IHC staining for BrdU in proximal colon of an S mouse at 125 days after TAM injection and a Cre-negative control (Cont). Scale bars: 50 μM. (**B**) Quantification of proliferating (BrdU-positive) cells per crypt from S (120–140 days after TAM, *n* = 6) and Cont mice (*n* = 5). Solid shapes represent the mean BrdU-positive cells per crypt for each mouse; smaller shapes represent individual values per crypt (15–59 crypts/mouse). **P = 0.0012 (Student’s *t* test, S vs. Cont). (**C**) Length of colon crypts from the mice in **B,** plus 1 additional AS mouse. Solid shapes represent the mean crypt length for each mouse; smaller shapes represent individual crypt lengths (12–30 crypts/mouse). *P = 0.0105 (Student’s *t* test, S vs. Cont). Scatter plots in **B** and **C** show mean ± SD. (**D**) Representative IHC staining for BrdU in proximal colon of A, AS, and Cont mice 35 days after TAM. Scale bars: 100 μM. (**E**) BrdU-positive cells as a percentage of total cells in proximal colon of A (*n* = 5), AS (*n* = 5), and Cont mice (*n* = 2) 33–37 days after TAM. *P = 0.045 (Student’s *t* test, AS vs. A). Data are shown as mean ± SD. (**F**) TUNEL assay in proximal colon of S, A, AS, and Cont mice 35 days after TAM. Arrows indicate apoptotic cells in S and Cont tissues. Scale bars: 20 μM. (**G**) TUNEL-positive apoptotic cells as a percentage of total cells in proximal colon of S (*n* = 8), A (*n* = 5), AS (*n* = 5), and Cont (*n* = 8) mice. *P = 0.01 (AS vs. A) and **P = 0.004 (S vs. Cont) from Student’s *t* test. Data are shown as mean ± SD.

Previous studies reported *Sox9* inactivation via Villin-Cre targeting in mouse intestine and colon epithelium led to increased proliferation and larger crypts (11, 24). In colon epithelium of S mice after our TAM-mediated gene targeting at both early (30–40 days after TAM) and later time points (120–140 days after TAM), we noted S crypts were elongated relative to Cont crypts (Figure 1; Figure 3, A–C; and Supplemental Figure 3), and we found increased cell proliferation in S versus Cont crypts (Figure 3, A and B, and Supplemental Figure 4). Colon crypts of A mice had extensive proliferation (Figure 3, D and E), but cell proliferation was reduced in AS epithelium relative to A mice (Figure 3, D and E). Proliferative changes can be offset by cell death, so we studied apoptosis. We found S and AS epithelium had reduced apoptosis relative to Cont epithelium and A epithelium, respectively (S vs. Cont and AS vs. A), whereas A epithelium had increased apoptosis compared with Cont epithelium (Figure 3, F and G). Our findings suggest the thickened colon mucosa resulting from combined *Apc* and *Sox9* inactivation relative to *Apc* inactivation may result chiefly from reduced apoptosis rather than enhanced cell proliferation.

### EMT induction in colon tumors with combined Apc and Sox9 inactivation.

**Figure 4:**
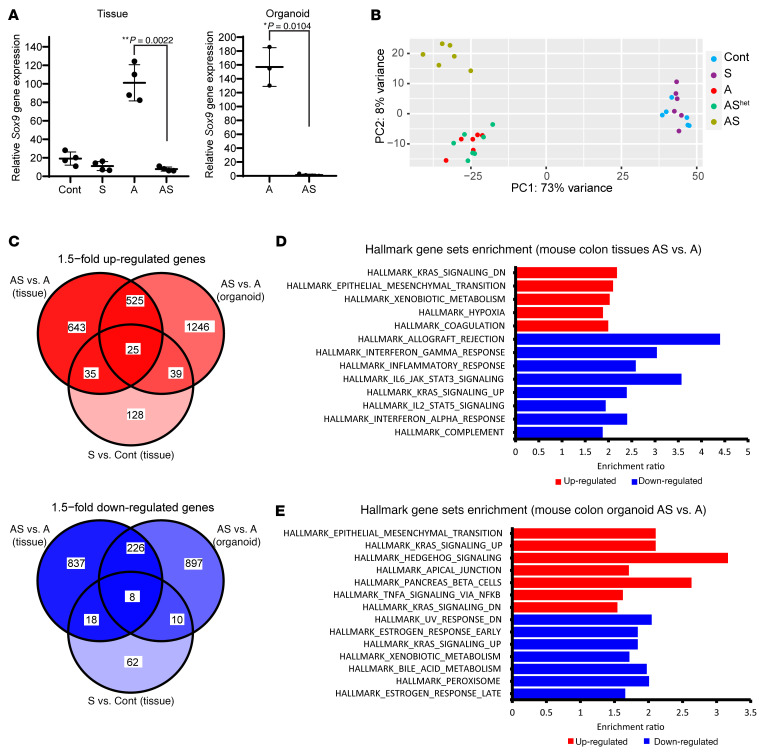
Differential gene expression in mouse primary colon tissues and organoids with *Sox9* inactivation. (**A**) *Sox9* gene expression was assessed by qRT-PCR in mouse proximal colon tissues from control mice (Cont) and S, A, and AS mice at 27–40 days after TAM dosing for gene targeting (*n* = 4 for each group). *Sox9* gene expression was also assessed by qRT-PCR in organoids derived from proximal colon of A (*n* = 3) and AS mice (*n* = 4). Gene expression was normalized to β-actin expression. **P = 0.0022 for colon tissue by Welch’s *t* test; *P = 0.0104 for organoids in AS versus A comparison; data are shown as mean ± SD. (**B**) Global gene expression analyses were performed with RNAs from colon tissues of Cont, S, A, AS^het^, and AS mice at 27–40 days following TAM treatment (*n* = 6 for each genotype except *n* = 7 for AS^het^). Principal component analysis showed colon tissue of AS mice had distinct global gene expression patterns compared with that in Cont, A, and AS^het^ mice. However, no separation was observed between S and Cont mice or between A and AS^het^ mice. (**C**) Venn diagram of differentially expressed genes in mouse colon tissues and organoids for the comparisons of AS versus A (tissues and organoids) and S versus Cont (tissues). Both upregulated (red) and downregulated (blue) genes induced by *Sox9* inactivation (fold change > 1.5, adjusted *P* ≤ 0.05) are shown. (**D** and **E**) Hallmark gene sets (FDR < 0.05) that are overrepresented in the upregulated (red) and downregulated (blue) genes in mouse colon tissues (**D**) and colon organoids (**E**) from AS versus A mice following TAM treatment.

To explore the molecular basis by which *Sox9* inactivation enhanced *Apc*-dependent tumorigenesis, we undertook global gene expression analyses on primary colon tissues from S, A, AS^het^, and AS mice. As a complementary approach to focus on gene expression changes specific to epithelial cells, we also studied organoids derived from colon epithelium of TAM-treated A and AS mice. TAM-induced *Sox9* inactivation in S and AS epithelium was confirmed by qRT-PCR (Figure 4A), consistent with loss of SOX9 protein nuclear staining on colon tissues from S and AS mice (Figure 1 and Supplemental Figure 3).

**Figure 5:**
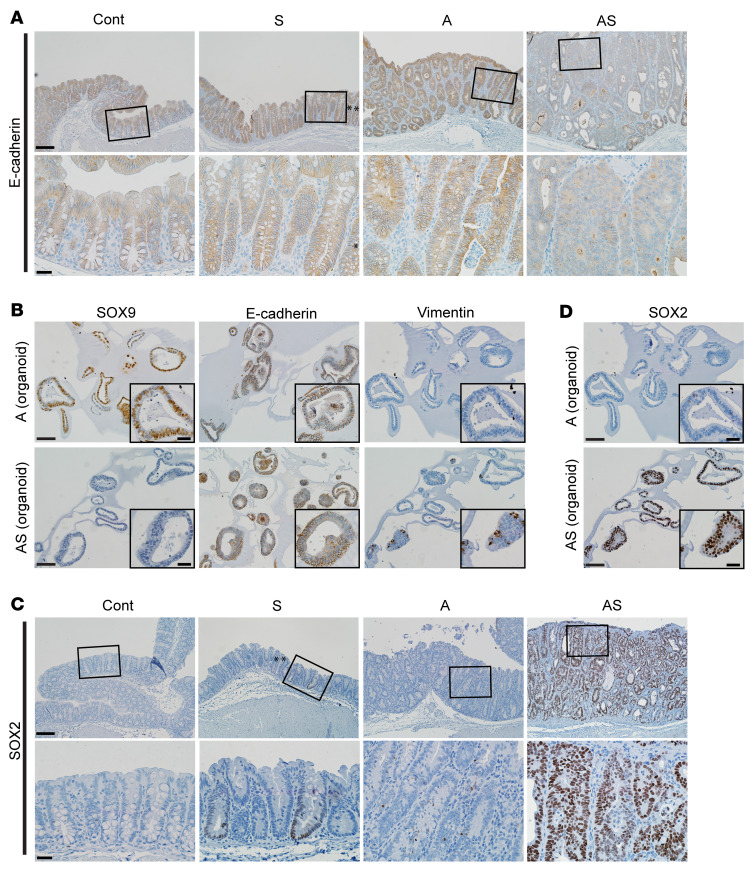
Reduced expression of E-cadherin and increased expression of SOX2 and vimentin in mouse colon epithelium with *Sox9* inactivation. (**A**) IHC staining for E-cadherin in proximal colon tissues of Cont, A, and AS mice at 35 days after TAM treatment, and an S mouse at 125 days after TAM treatment. Representative images with low (top row) and high (bottom row) magnifications for each mouse are shown. Asterisks denote SOX9-positive crypts that show incomplete gene targeting in the S mouse. (**B**) Representative photomicrographs of IHC staining for SOX9, E-cadherin, and vimentin in mouse colon organoids derived from A and AS mice after TAM treatment. (**C**) IHC staining for SOX2 in mouse proximal colon tissues of Cont, A, and AS mice at 31 days after TAM treatment, and an S mouse at 70 days after TAM treatment. (**D**) Representative photomicrographs of IHC staining for SOX2 in mouse colon organoids as described in **B**. Scale bars for **A** and **C**: 100 μM for low magnification image (top); 20 μM for high magnification images (bottom). Scale bars for **B** and **D**: 50 μM; 20 μM for insets.

Principal components analysis showed AS colon tissues had global gene expression distinct from Cont, A, or AS^het^ tissues (Figure 4B). Colon tissues from S and Cont mice had similar global gene expression, and A and AS^het^ tissues exhibited essentially indistinguishable global gene expression (Figure 4B). A clear distinction in global gene expression was also observed in AS versus A organoids (Supplemental Figure 5A). When comparing gene expression in A versus AS colon tissues, we identified 2,317 differentially expressed genes (1,228 upregulated and 1,089 downregulated in AS epithelium with a fold change > 1.5, adjusted *P* ≤ 0.05). A few of these genes were also altered in the same direction in S tissues (60 upregulated and 26 downregulated in S versus control colon tissues, as illustrated in Figure 4C). In A versus AS organoids, we observed many differentially expressed genes (1,835 upregulated and 1,141 downregulated in AS vs. A organoids), and multiple genes (550 upregulated and 234 downregulated genes) had similar expression patterns in colon tissues and organoids of a given genotype (Figure 4C). Notably, for upregulated genes, EMT was the Hallmark gene set with second-best enrichment in AS versus A mouse colon tissues and the best enrichment in organoids (Figure 4, D and E). Comparison of AS and A organoids showed upregulation of more EMT-related genes (48 out of 200 EMT genes) relative to colon tissues (29 out of 200), with genes for 2 well-known mesenchymal markers, *Cdh2* and *Vim*, increased by 14-fold and 5-fold, respectively, in AS versus A organoids (Supplemental Table 1). Moreover, AS organoids also had significantly increased levels of transcription factors known to induce EMT — *Snai1* (by 4-fold) and *Twist1* (by 48-fold) as well as a trend for *Snai2* expression (by 12-fold, approaching significance) — relative to A organoids (Supplemental Table 1). In line with the augmented EMT gene signature in AS versus A colon tissues, we noted a substantial decrease in membrane staining for the epithelial E-cadherin protein in the AS colon epithelium relative to the prominent membrane staining for E-cadherin observed in cells in Cont, S, or A epithelium (Figure 5A). Although only a fraction of AS organoid cells showed reduced E-cadherin membrane staining compared with the stronger and more uniform membrane E-cadherin staining in A organoids, increased expression of vimentin protein (a mesenchymal marker) was seen in AS organoids relative to A organoids (Figure 5B).

### Activation of the stemness factor SOX2 in Sox9-defective colon cancers is linked to EMT induction and an enhanced metastatic phenotype.

We found the *Sox2* gene, which encodes a transcription factor involved in cell fate specification and stem cell phenotypes, was among the most significantly upregulated genes in *Sox9*-mutant colon tissues and organoids compared with those with intact *Sox9*, including 115-fold activation in AS tissues versus A tissues and 4-fold activation in AS^het^ versus A tissues; 10-fold activation in S tissues compared with Cont tissues; and 38-fold activation in AS organoids compared with A organoids (Supplemental Figure 6B). We confirmed strong nuclear expression of the SOX2 protein in S and AS mouse primary colon tumors (Figure 5C) and organoids (Figure 5D) where the *Sox9* gene is inactive, whereas only approximately 5% of cells in tissues from A mice showed weakly positive SOX2 staining (Figure 5, C and D). Besides its role in embryonic development and tissue homeostasis, SOX2 has garnered attention in cancer (25, 26), due to its amplification or overexpression in some human cancers and evidence SOX2 may regulate cancer stem cell properties and tumorigenic phenotypes, perhaps in part via activation of EMT in cancer (26).

We also pursued studies of SOX2 protein expression in colon tumors arising in an independent mouse CRC model based on mutations in 3 CRC-associated genes — heterozygous mutations in the *Apc* gene and the *Kras* oncogene together with biallelic defects in the *Trp53* tumor suppressor genes (previously described as AKP^270/fl^ mice and abbreviated here as AKP mice) (27). We found SOX2 was expressed in 13% of 92 independent colon tumors obtained from 10 AKP mice, but not in adjacent normal colon epithelium, and SOX2 expression was linked to high tumor grade (*P* < 0.0001) and invasiveness (*P* < 0.0001), suggesting a role for increased SOX2 in mouse CRC progression (Supplemental Figure 7 and Supplemental Table 2). Additionally, some liver and lymph node metastases in AKP mice strongly expressed SOX2 (Supplemental Figure 8 and Supplemental Table 2).

To strengthen the case for the functional role of *Sox9* loss to increased SOX2 levels and tumor progression, we studied how *Sox9* genetic inactivation affected the AKP model and the tumors arising (termed AKPS tumors). All AKPS primary colon tumors were SOX2 positive and displayed high tumor grade and invasive features (Supplemental Figure 7 and Supplemental Table 2). Furthermore, *Sox9* deletion increased the incidence of metastasis, with 6 of 11 AKPS mice harboring lymph node and liver metastases, and 1 of 11 mice having a lung metastasis, compared with only 2 of 11 AKP mice with lymph node metastases and 1 of 11 AKP mice with liver and lung metastases (Supplemental Table 3). All detected metastatic lesions in AKPS mice were SOX2 positive (Supplemental Figure 8 and Supplemental Table 2), strongly linking SOX2 expression to enhanced metastasis in the AKPS tumor model.

We then addressed how SOX2 overexpression altered the phenotypes of 2 independent mouse colon cancer cell lines (AKP1 and AKP2) derived from AKP tumors. Although SOX2 overexpression did not further enhance in vitro migration and invasion in the AKP cancer cell lines (data not shown), SOX2 ectopic expression markedly increased expression of the vimentin and N-cadherin mesenchymal proteins and the EMT-inducing transcription factor TWIST1 in both AKP lines as well as the SNAI2 (also known as Slug) transcription factor in the AKP1 line (Supplemental Figure 9). These data, together with our in vivo findings showing *Sox9* inactivation enhanced *Sox2* expression and EMT, indicate *Sox9* inactivation likely promotes EMT in part via increased SOX2 expression. Although we found Cre-mediated *Sox9* gene deletion could be detected at 2 days after TAM treatment (Supplemental Figure 10A), increased SOX2 protein expression was not found in AS epithelium until 8 days after TAM. SOX2 induction began at the crypt base and showed a crypt base to lumen gradient at least through day 15 (Supplemental Figure 10A). By day 31 after TAM, robust SOX2 expression was observed throughout AS crypts (Supplemental Figure 10B). The temporal and spatial relationships for *Sox9* inactivation and SOX2 induction in colon crypts are consistent with the proposal that *Sox9* inactivation likely leads to induction of SOX2 expression in *Apc*-mutant colon epithelium by altering cell fate rather than by directly affecting *Sox2* transcriptional regulation and/or protein expression. Because SOX2 has been implicated in maintaining stemness (25), upregulation of SOX2 in AS colon epithelium may enforce a stemness program.

We also saw increased expression of other stem cell markers in AS colon epithelium, such as *Msi2*, *Bmi1*, and *Smoc2* (comparing AS with A epithelium, Supplemental Table 4). Further, *Sox9* inactivation in *Apc*-mutant epithelium was linked to reduced intestinal differentiation, as reflected by decreased gene expression of intestinal differentiation markers for Paneth, secretory, and absorptive cell lineages in AS versus A mice (Supplemental Table 4). *Apc* inactivation in A mice led to generation of Paneth-like cells (marked by lysozyme staining), which are normally present in the small intestine but are absent in mouse colon epithelium, as well as a reduction in goblet cells (as detected by Alcian blue staining), and continued expression of the intestinal differentiation marker villin (Supplemental Figure 11). In AS colon epithelium, we saw a marked reduction in ectopic Paneth-like cells, goblet cells, and villin expression (Supplemental Figure 11). Colon epithelium in AS^het^ mice displayed lysozyme, Alcian blue, and villin staining similar to A mice. Our findings further support a tumor suppressor role for *Sox9* in *Apc* mutation–dependent tumorigenesis, with combined *Sox9* and *Apc* biallelic inactivation leading to lesions with high-grade dysplasia and invasion typical of carcinoma, as well as an apparent block to intestinal differentiation. Our data on the role of endogenously expressed SOX9 in promoting cell differentiation in vivo contrast markedly with in vitro findings (17).

### Sox9 inactivation has nominal effects on the Wnt signaling pathway in colon epithelium.

The *Sox9* gene was previously shown to be regulated by the Wnt/β-catenin signaling pathway (6, 23), and SOX9 ectopic overexpression can suppress Wnt/β-catenin signaling in cell culture–based studies (9, 13, 28). We sought to determine whether and how loss of endogenous *Sox9* function might affect the Wnt/β-catenin signaling pathway. Inactivation of *Sox9* on its own in colon epithelium (S mice) did not alter membrane β-catenin staining patterns relative to Cont mice (Figure 1 and Supplemental Figure 3, A and B). As expected from *Apc* inactivation, A and AS colon epithelium had strong cytoplasmic and nuclear β-catenin expression (Figure 1). Immunoblot analysis of A and AS tissues showed results in line with the IHC findings, with increased levels of the active, dephosphorylated form of β-catenin in A and AS mice relative to Cont and S mice (Supplemental Figure 6A).

We then studied expression of 31 other Wnt/β-catenin target genes in mice with intact versus inactive *Sox9*. In A tissues, among the 31 Wnt pathway genes studied (28 previously known to be upregulated and 3 downregulated by β-catenin–dependent Wnt signaling), we found that 23 of the 28 expected upregulated genes were indeed upregulated, and all 3 known downregulated genes were downregulated (Supplemental Figure 6B). S tissues showed Wnt target gene expression like Cont tissues, except for slight induction of *Notum* and *Sox2* (Supplemental Figure 6B). In AS tissues, Wnt target gene expression was like that in A tissues (Supplemental Figure 6B; 24 upregulated and 3 downregulated), except for strong induction of *Sox2* gene expression in AS tissues and minor effects on a few other target genes (e.g., *Fgf20, Sox17, Dkk1*). AS^het^ tissues did not show demonstrable changes in expression of Wnt target genes relative to A tissues, except for a 4-fold increase in *Sox2* expression (Supplemental Figure 6B). Our data indicate loss of SOX9 function in mouse colon epithelium affects expression of a very limited number of Wnt pathway target genes and likely through gene-specific mechanisms rather than by broadly affecting β-catenin levels and β-catenin/TCF–regulated transcription.

### SOX9 expression loss in human CRCs is linked to higher tumor grade and epigenetic silencing.

**Figure 6:**
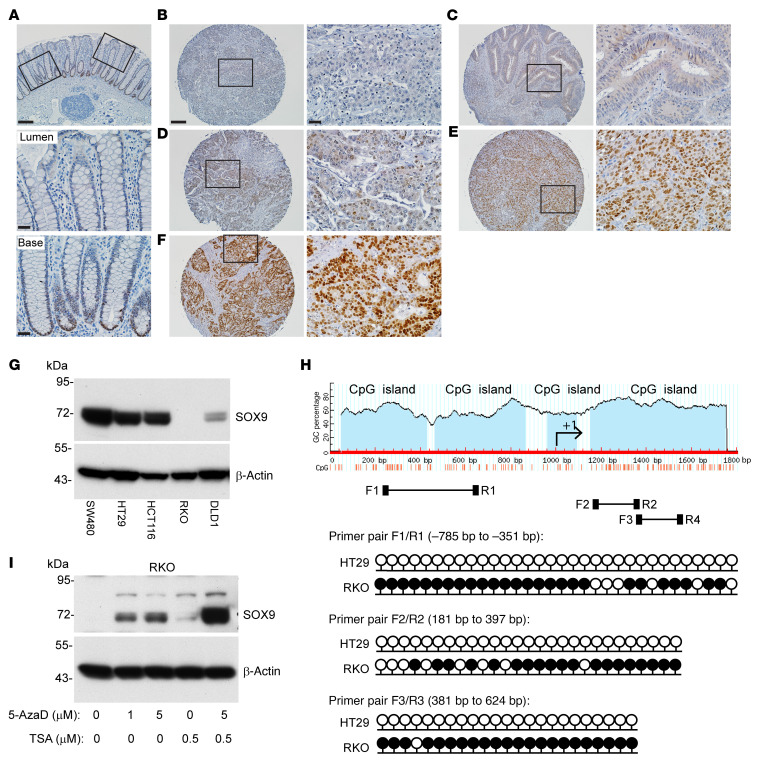
SOX9 protein expression and *SOX9* gene methylation status in CRC primary tissues and selected cell lines. (**A**) Representative photomicrographs of IHC staining for SOX9 in normal human colon tissue showing nuclear SOX9 expression in crypt base cells. The image is shown with low magnification for the entire colon crypts; boxed areas for lumen and crypt base regions are also shown as high magnification (middle and bottom panels). (**B**–**F**) Representative photomicrographs of IHC staining for SOX9 in CRCs in a human tissue microarray, with different IHC scores of (**B**) 0, (**C**) +/–, (**D**) 1+, (**E**) 2+, and (**F**) 3+. The images with low magnification (left) and their corresponding boxed areas with high magnification (right) are displayed for each CRC. Scale bars: 100 μM (low magnification), 20 μM (high magnification). (**G**) Immunoblot analysis for SOX9 in human colon cancer cell lines, with β-actin as a loading and transfer control. (**H**) Diagram of CpG islands in the *SOX9* promoter and first exon, and locations of primers for bisulfite sequencing (F1/R1, F2/R2, F3/R3). Lollipop diagrams of bisulfite sequencing of *SOX9* at the indicated regions (relative to the +1 transcription start site) in HT29 and RKO cells are shown with black lollipops for methylated CpGs and white for unmethylated. At least 5 clones were sequenced for each primer pair, and methylation of an individual CpG dinucleotide was confirmed in at least 2 clones. (**I**) RKO cells were treated with vehicle (0) or treated with 1 μM or 5 μM 5-Aza-2′-deoxycytidine (5-AzaD) for 3 days to induce DNA demethylation. During the third day of treatment with vehicle or 5-AzaD, cells were further incubated with vehicle or 0.5 μM trichostatin A (TSA) for 24 hours. SOX9 expression in the cells was assessed by immunoblot, with β-actin as a loading and transfer control.

*SOX9* somatic mutations are found in about 5%–10% of human CRCs, and most mutations are frameshift or nonsense, suggesting a possible tumor suppressor role of *SOX9* in CRCs (7–9, 19). However, almost all CRCs with *SOX9* coding region mutations retain prominent SOX9 protein expression, as the nonmutated *SOX9* allele is expressed (8, 29). To further evaluate the role of *SOX9* in human CRCs, we examined SOX9 protein expression by IHC in relation to clinicopathological factors using a human colon tumor primary tissue microarray (TMA). In normal colon tissue, SOX9 expression was only found in the nucleus of epithelial cells at the crypt base (Figure 6A). Of 171 human colon adenocarcinomas studied, 70% of the tumors showed strong nuclear expression of SOX9 throughout the neoplastic epithelium (Figure 6, B–F). On the other hand, we found 21% of the tumor samples studied showed absent or negligible SOX9 staining (Figure 6, B and C, and Supplemental Table 5). Loss of SOX9 protein expression was associated with increased tumor-grade. The odds of SOX9 loss (SOX9_Score = 0) in patients with low-grade tumors was only 0.23 times as likely as in patients with high-grade tumors (OR = 0.23 in univariate analysis of factors associated with loss of SOX9 protein expression, *P* = 0.001 by Fisher’s exact test; see Table 1 and Supplemental Table 5). Correlations of SOX9 loss for other clinicopathologic factors studied were not statistically significant (Table 1).

We assessed SOX9 protein expression by immunoblot analysis in 5 human CRC cell lines. High SOX9 protein expression was found in 3 cell lines (SW480, HCT116, and HT29), with substantially reduced SOX9 expression in DLD1 and complete loss of SOX9 expression in RKO (Figure 6G). DNA methylation of *SOX9* gene upstream regions has been documented in several cancer types, including bladder cancer (30), cervical cancer (31), and melanoma (32), and SOX9 expression can be silenced by *SOX9* DNA methylation in bladder (30) and melanoma models (32). We compared the *SOX9* DNA methylation status in SOX9-high HT29 cells and SOX9-negative RKO cells using bisulfite sequencing. We studied the DNA methylation status of a cluster of 32 CpGs across a 434-bp region (using primer pair F1/R1), a cluster of 27 CpGs across a 217-bp region (using primer pair F2/R2), and a cluster of 27 CpGs across a 244-bp region (using primer pair F3/R3) from 3 CpG islands, all located within the region approximately 1,000 bp upstream of the *SOX9* transcriptional start site to exon1 (Figure 6H). The majority of CpGs in all 3 regions of the *SOX9* promoter were methylated in RKO cells and unmethylated in HT29 cells (Figure 6H). To confirm *SOX9* expression is regulated by DNA methylation, we tested the DNA methyltransferase inhibitor 5-Aza-2′-deoxycytidine (5-AzaD) in RKO cells and found SOX9 expression was restored by treatment with 5-AzaD in a dose-dependent manner (Figure 6I). The combination of 5-AzaD and the histone deacetylase inhibitor trichostatin A induced substantially more SOX9 expression than either treatment alone (Figure 6I), suggesting promoter DNA hypermethylation and histone deacetylation could each play a role in silencing SOX9 expression. To further investigate the role of DNA methylation in *SOX9* gene expression in CRC pathogenesis, we downloaded methylation (HM450) beta-values for the *SOX9* gene (for genes with multiple methylation probes, we selected the probe most anticorrelated with expression) and *SOX9* mRNA expression values (RNA-Seq V2 RSEM) for 353 patients with CRC from The Cancer Genome Atlas (TCGA) via cBioPortal. A correlation plot of mRNA expression and DNA methylation levels for *SOX9* showed *SOX9* expression inversely correlated with its DNA methylation, with a Spearman’s correlation coefficient of *r* = –0.34 and *P* = 9.46 × 10^–11^ (Supplemental Figure 12). Taken together, our data indicate DNA methylation of the *SOX9* promoter region likely underlies *SOX9* epigenetic silencing in the roughly 20% of human CRCs lacking SOX9 protein expression.

### Low SOX9 gene expression is correlated with earlier onset, lymphatic invasion, and poor outcome in patients with CRC.

**Figure 7:**
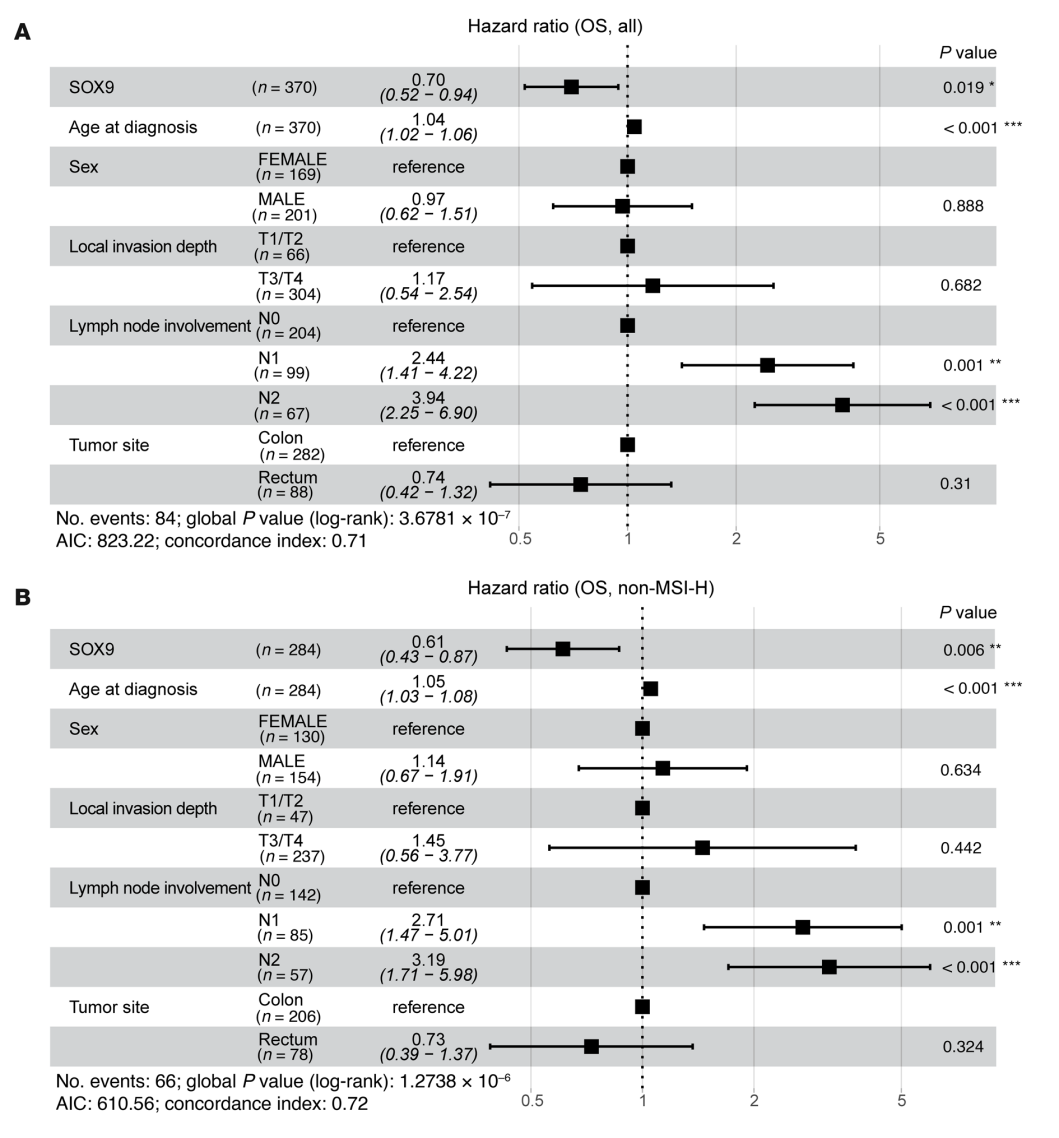
Multivariate analysis of overall survival in patients with CRC. The forest plots display the results of multivariate Cox proportional hazards models for OS, including *SOX9* gene expression (log_2_-transformed), age at diagnosis, sex, local invasion depth, lymph node involvement, and tumor site as covariates, in all patients with CRC from the TCGA Colon and Rectal Cancer (COADREAD) cohort (*n* = 370) (**A**), and patients without MSI-H from the same cohort (*n* = 284) (**B**). The square represents estimated HR, and the length of the horizontal line represents the 95% CI for the HR of each covariate. *P* values for individual covariates were obtained using the Wald test (column on the far right). Statistical significance is indicated by asterisks: *P < 0.05; **P < 0.01; ***P < 0.001.

To further investigate whether *SOX9* gene expression is associated with survival outcomes in patients with CRC, we performed survival analyses using data from the TCGA Colon and Rectal Cancer (COADREAD) cohort. Associations between *SOX9* gene expression and 3 types of survival outcomes — overall survival (OS), disease-specific survival (DSS), and progression-free interval (PFI) — were evaluated using multivariate Cox proportional hazards models, adjusting for age at diagnosis, sex, local invasion depth, lymph node involvement, and tumor site. For OS, lower *SOX9* expression (HR = 0.70; 95% CI: 0.52–0.94; *P* = 0.019), older age at diagnosis (HR = 1.04; 95% CI: 1.02–1.06; *P* < 0.001), and greater lymph node involvement (N2 vs. N0: HR = 3.94; 95% CI: 2.25–6.90; *P* < 0.001) were identified as significant independent predictors of worse OS (Figure 7A). For DSS, lower *SOX9* expression showed a near-significant association (HR = 0.70; 95% CI: 0.47–1.00; *P* = 0.081) with poorer outcomes, while more lymph node involvement (N2 vs. N0: HR = 6.04; 95% CI: 2.55–14.30; *P* < 0.001) was significantly associated with worse DSS (Supplemental Figure 13A). However, there was insufficient evidence to conclude that *SOX9* expression significantly affected PFI (Supplemental Figure 13B). Microsatellite instability-high (MSI-H) is a molecular phenotype caused by defects in the DNA mismatch repair system and is detected in approximately 15% of CRCs. Both high and low SOX9 expression levels have previously been reported to associate with MSI (33, 34), though one study found no association between MSI-H and SOX9 expression in patients with stage II CRC (19). In our study, using the same TCGA CRC cohort (339 patients with available MSI status [ref. 35]), we found that low *SOX9* expression (10 MSI-H CRCs out of 49 CRCs with low *SOX9* expression) was not significantly associated with MSI-H (*P* = 0.2798, Fisher’s exact test). Among patients without MSI-H, the multivariate Cox model indicated that lower *SOX9* expression remained significantly associated with poorer OS (HR = 0.61; 95% CI: 0.43–0.87; *P* = 0.006) (Figure 7B). A likelihood ratio test comparing models with and without an interaction term for MSI status and *SOX9* expression showed that MSI status did not significantly modify the effect of *SOX9* expression on OS, DSS, or PFI (data not shown).

To further examine the relationship between *SOX9* expression and various clinicopathological factors as well as other gene expressions in the same TCGA CRC patient cohort, we first sought a prognostically meaningful cutoff to dichotomize *SOX9* gene expression. We applied the surv_cutpoint function from the “survminer” R package (based on OS) to identify an optimal cutoff in Kaplan-Meier curves that maximally separated the cohort into distinct prognostic groups. This analysis yielded 11.66 (log_2_ scale) as the optimal cutoff for *SOX9* gene expression, stratifying patients into low (≤11.66, *n* = 56) and high (>11.66, *n* = 320) *SOX9* expression groups (Supplemental Figure 14). A similar cutoff (11.65) was identified via an alternative regression tree method (data not shown). As shown in Supplemental Figure 15, Kaplan-Meier curves based on this optimal cutoff indicated that the patient group with low *SOX9* gene expression had significantly decreased OS, DSS, and PFI compared with the group with higher *SOX9* gene expression. Furthermore, we found low *SOX9* gene expression was significantly associated with an earlier age at diagnosis (*P* = 0.0089), more lymphatic invasion (*P* = 0.0002), and more lymph node involvement (*P* = 0.0028) (Table 2). The CRCs with low *SOX9* expression also tended to have more venous invasion (*P* = 0.069) and higher tumor stages (*P* = 0.068) (Table 2). We obtained RNA-Seq data for the same TCGA CRC patient cohort and compared gene expression in patients with low *SOX9* gene expression (*n* = 56) versus patients with high *SOX9* gene expression (*n* = 320). Our differential gene expression analysis (FDR-adjusted *P* < 0.05, fold change >1.5 in either direction) yielded 3,896 differentially expressed genes (DEGs, 3,152 upregulated and 744 downregulated in patients with low *SOX9* gene expression, Supplemental Figure 16). Our gene enrichment testing with the DEGs above against curated gene sets from MSigDB identified 12 gene sets for the list of genes upregulated and 4 gene sets for the list of genes downregulated in CRCs with low *SOX9* expression (Table 3). Notably, the best enrichment for upregulated genes was EMT (108 of 200 genes, *P* = 3.1439 × 10^–29^), including *CDH2,* *FN1, TGFB1,* and *VIM*. Furthermore, we observed the EMT-inducing transcription factors *SNAI2,* *TWIST1, TWIST2, ZEB1,* and *ZEB2* were also significantly higher in CRCs with low *SOX9* expression (Supplemental Table 1). Consistent with the aggressiveness of CRCs with low *SOX9* expression, enriched pathways for our upregulated genes in these tumors also included KRAS signaling and angiogenesis (Table 3). *Sox9* inactivation caused a modest increase in phospho-ERK activity in *Apc*-deficient mouse colon epithelium (Supplemental Figure 17), indicating a possible role for SOX9 in regulating MAPK signaling. Interestingly, human CRCs with low *SOX9* expression showed some different mutation frequencies of selected recurrently mutated genes compared with CRCs with intact/high *SOX9* expression. Low *SOX9* expression status was associated with reduced frequencies of mutations in *APC* (*P* < 0.0001), *KRAS* (*P* = 0.01), and *SOX9* (*P* = 0.0041), and higher frequency of mutations in *CTNNB1* (*P* = 0.0069) and *AXIN1* (*P* = 0.0199) (Supplemental Table 6). Overall, our findings from in-depth analysis of the TCGA data bolster the view that *SOX9* functions as a tumor suppressor in a notable subset of human CRCs, with loss of *SOX9* expression and function promoting tumor progression and invasion and poorer patient outcomes.

## Discussion

SOX9 has been implicated as a key factor in cell fate determination in development and in adult tissues (36). The role of SOX9 in cancer remains incompletely understood (14–16). Both tumor suppressor and oncogenic functions of SOX9 have been suggested, including in CRC (14, 16). We pursued studies of loss of SOX9 function in vivo because apparent ambiguities and uncertainties with prior findings may reflect SOX9 overexpression or insufficiently controlled gene knockdown approaches in tissue culture studies. We comprehensively detailed cellular and molecular alterations in mouse colon epithelium with inactivation of both *Sox9* and *Apc* versus *Apc* inactivation alone. In contrast to the low-grade dysplasia observed in *Apc*-deficient colon epithelium, epithelium with both *Apc* and *Sox9* inactivation exhibited pronounced thickening, a notable increase in crypt fission and branching, and prominent nuclear atypia and multifocal cribriform glandular architecture, which are characteristics of high-grade neoplasia. Additionally, colon lesions with *Apc* and *Sox9* inactivation showed reduced apoptosis and more invasive features compared with lesions with only *Apc* inactivation. Moreover, biallelic inactivation of *Sox9* in an independent mouse colon tumor model (AKP mice) significantly increased the burden of tumors that were high tumor grade, invasive, and metastatic. Our findings from the mouse models recapitulate features seen in patients with CRC, where low SOX9 expression correlated with higher tumor grade, increased lymphatic invasion, and poorer patient survival.

Our findings provide information about the role of *SOX9* as an epigenetically silenced tumor suppressor gene in the roughly 20% of human CRCs with markedly reduced or absent SOX9 expression. The proposal that *SOX9* might function as an oncogene in human CRC is based on indirect evidence. To the best of our knowledge, there are no mutational data, such as high-copy gene amplification, gene fusions and translocations, or recurrent localized missense mutations that establish clear-cut gain-of-function defects for *SOX9* in CRCs, which are the types of genetic data needed to support a role for SOX9 as an oncogene in CRC biology, akin to the genetic evidence that exists for known oncogenes, such as *MYC*, *KRAS*, and *BRAF*. Also, the functional studies offered as evidence that SOX9 might function as an oncogene emphasize cell line–based efforts to reduce endogenous SOX9 expression and then study in vitro cell proliferation and differentiation properties of the cells.

The human CRCs in the TCGA series with low/absent SOX9 gene expression have a lower *APC* mutation rate (48%) than the *APC* mutation rate (80%) seen in CRCs with strong *SOX9* expression. These findings presumably indicate that selection for *SOX9* silencing is more often present in, but is not restricted to, the setting when the *APC* gene is functionally intact. On the other hand, CRCs that have low expression of *SOX9* have higher mutation rates in genes for other Wnt pathway components (e.g., *CTNNB1* and *AXIN1*). We found *Sox9* inactivation had negligible effects on the Wnt signaling pathway, as evidenced by the lack of observed effects on β-catenin levels and localization or consistent effects on Wnt target genes when *Sox9* was inactivated in colon epithelium. Overall, although the human CRC mutational data suggest *SOX9* inactivation may interact with *APC* mutation–dependent tumorigenesis, the apparent relationship between *SOX9* loss and *APC* mutation status in CRCs does not appear to reflect a functional connection between SOX9 expression status and β-catenin/TCF–dependent transcriptional activity.

Our in vivo results on effects of *Sox9* inactivation on stem cell markers and differentiation are at odds with some tissue culture–based results. For instance, in published work, downregulation of endogenous SOX9 expression via knockdown approaches in human CRC cell lines and in neoplastic mouse organoids was reported to impair cell growth and induce differentiation, accompanied by reduced expression of stem cell markers (mainly *Lgr5*, *Ascl2*, *Prom1*, and *Lrig1*) (17). The prior findings were interpreted by the authors as evidence that SOX9 overexpression blocked differentiation in CRC cells by promoting a stem cell–like program (17). In contrast, in our study, in mouse colon epithelium with combined *Apc* and *Sox9* inactivation, we found significantly increased expression of stem cell markers (e.g., *Msi1, Msi2, Bmi1,* and *Sox2)* and significantly reduced expression of intestinal differentiation markers for Paneth cells, secretory cells, and absorptive cells compared with *Apc*-defective colon epithelium. When we compared the *Apc* and *Sox9*-defective colon epithelium to normal colon epithelium, we found significantly increased expression of most stem cell markers, including *Lgr5*, *Lrig1*, *Msi1, Msi2, CD44*, *Bmi1,* and *Sox2*.

In addition, some conflicting results have been reported in previous studies of *Sox9* defects in *Apc* mutation–induced tumorigenesis in other genetically engineered mouse models. One study reported *Sox9* inactivation in mouse intestinal epithelium increased the number of large adenomas and overall tumors in *Apc^min/+^* mice, but there was no description of whether *Sox9* inactivation affected biological characteristics of the adenomas arising (37). A different study employed an alternative mouse genetic model to the *Apc^min/+^* mouse model or our models, where the authors utilized an *Lgr5* promoter–driven Cre transgene to concurrently inactivate *Apc* and/or *Sox9* in mouse small intestine and colon epithelium. The authors reported *Sox9* inactivation prevented *Apc*-mutation–induced adenoma formation, reduced an aberrant stem cell–like signature, and prolonged survival compared with *Apc* inactivation on its own (20). Notably, the authors did not detect any adenomas with the expected Cre-mediated biallelic inactivation of *Sox9*, which led the authors to conclude *Sox9* inactivation blocks *Apc* mutation–induced adenoma initiation. These findings and conclusions with the Lgr5-Cre model stand in direct contrast to those in our study and the *Apc^min/+^* mouse study noted previously (37), where biallelic *Sox9* inactivation can clearly be demonstrated in adenomas arising and where the findings implicate *Sox9* function as a tumor suppressor gene. Another key difference in our study is unlike *Apc^min/+^* mice and *Lgr5-CreER* *Apc^fl/fl^* mice that develop adenomas chiefly in the small intestine (38, 39), lesions in our mouse models arise in the colon and cecum (23), which more closely reflects human CRC.

To study how the SOX9 protein functions in *Apc* mutation–dependent tumorigenesis, we conducted gene expression profiling. We found colon tissues with concurrent inactivation of *Sox9* and *Apc* exhibited an enhanced EMT signature compared with epithelium with *Apc* deletion alone. An enhanced EMT signature was also observed in organoids with combined inactivation in *Apc* and *Sox9* and in patients with CRC with low *SOX9* gene expression. Consistently, compared with cells with *Apc* deletion only, we found decreased protein expression of the epithelial E-cadherin protein in colon epithelium with deletion of *Apc* and *Sox9*, and increased expression of vimentin, a mesenchymal protein, in the *Apc*-defective and *Sox9*-defective organoids. While the concept of an EMT has often been viewed as a binary switch from one state to the other, it is now evident epithelial cells can persist in a series of metastable, intermediate states with expression of selected epithelial (E) and mesenchymal (M) markers. Cells in these states can exhibit mixed E/M features and transition between intermediate E/M phenotypic states arrayed along the epithelial–mesenchymal spectrum. Various terms such as partial EMT, hybrid E/M status, and EMT continuum have been used to describe this plasticity. Recently, EMT researchers proposed the term epithelial-mesenchymal plasticity (EMP) for the dynamic cell states (40). In our study, despite marked reduction in membrane E-cadherin expression in colon epithelium with combined *Apc* and *Sox9* inactivation, weak membrane staining of E-cadherin persisted, and expression of the N-cadherin mesenchymal marker was not found, suggesting *Sox9* inactivation may trigger a partial EMT. Supporting this notion, we observed a modest increase in gene expression for a partial EMT marker Tenascin C (*Tnc*) and a decrease in gene expression of the epithelial marker *Epcam* in *Sox9*-defective colon epithelium compared with normal or *Apc*-defective epithelium. Partial EMT has been linked to increased invasiveness and metastatic potential in skin and breast cancer models (41, 42), and hybrid E/M states are enriched in circulating tumor cells released by primary breast and lung cancers and their metastases, suggesting phenotypic plasticity may provide cancer cells with better adaptability for survival (43, 44).

In our study, *Sox2* emerged as one of the most prominently upregulated genes in *Sox9*-defective colon epithelium. This observation aligns with other findings indicating a pivotal role of SOX2 in cancer progression and metastasis. Knockdown of SOX2 in human CRC cells has been reported to impair cancer cell stemness, in vitro cell migration and invasion, and metastasis in a mouse model (45, 46). Furthermore, suppression of SOX2 has been shown to promote a mesenchymal-epithelial transition (i.e., the reverse of EMT) and results in a significant reduction in the expression of key genes associated with EMT (45, 46). Other studies in other types of cancer cells have demonstrated overexpression of SOX2 promotes EMT (47–50). Our study with mouse colon cancer cell lines also clearly showed that overexpression of SOX2 led to increased expression of mesenchymal markers (vimentin and N-cadherin) and EMT-inducing transcription factors (e.g., TWIST1), supporting its role in promoting EMT in colon cancer. In patients, elevated SOX2 expression in CRC has been associated with advanced tumor grade, TNM (tumor, node, metastasis) stage, metastasis, and poor patient survival (45, 46, 51). In line with these studies, our own investigation clearly illustrated that tumors expressing elevated levels of SOX2 tended to exhibit high-grade characteristics, increased invasiveness, and a greater propensity for metastasis in mouse CRC models. Furthermore, our results indicated biallelic inactivation of *Sox9* in the A or AKP models was associated with higher tumor grades and enhanced invasiveness and metastasis. *Sox9* inactivation was notably correlated with the increased expression of SOX2, suggesting SOX9 inactivation plays a role in promoting tumor progression in part by SOX2 upregulation.

Previous work suggested SOX2 regulates tumorigenic features and EMT processes by modulation of MMP2 and MMP9 activity in human CRC (46) and ovarian cancer cells (47). It was also suggested SOX2 mediates these effects by modulating Wnt pathway signaling and β-catenin levels in cancer cells (46, 49, 50). However, our in vivo studies showed differences from cell line–based studies. Although biallelic *Sox9* inactivation in *Apc*-defective mouse colon epithelium significantly upregulated expression of *Sox2* and *Mmp9*, it did not induce significant changes in *Mmp2* expression or β-catenin levels in comparison to colon epithelium with *Apc* inactivation alone. Also, as noted above, we observed no consistent and significant changes in the expression of 31 independent Wnt target genes following *Sox9* inactivation, despite clear increases in SOX2 levels when *Sox9* was inactivated. This discrepancy between our in vivo findings and published cell line studies suggests the role of SOX2 in governing cell migration, invasion, and EMT may be strongly influenced by the specific cellular context. SOX2 has also been reported to promote maintenance of cancer stem cell (CSC) phenotypes, including in CRC (25, 26). Phenotypes of CSCs include decreased adhesion, increased CSC marker expression and asymmetric cell division, enhanced chemoresistance, and higher tumorigenicity in transplantation models (52, 53).

In closing, to our knowledge our study is among the first to link SOX9 loss to enhanced tumor aggressiveness and progression in CRC development in in vivo models and in patients. Furthermore, we have identified the upregulation of SOX2 as a potentially critical mechanism downstream of SOX9 loss, likely contributing to the observed EMT changes, CSC phenotypes, and tumor progression. Our findings and the differences in our work relative to previously published studies highlight the importance of studying cancer-associated genes in the in vivo setting and relating those findings to biological and clinical features in human cancer. The role of SOX9 in colon tumorigenesis is likely context dependent, with both the tumor’s origin and overall genotypic and epigenetic status influencing SOX9 tumor suppressor function. Finally, our study provides support for the view that EMT and stemness phenotypes contribute to tumor progression and metastasis in vivo in CRCs that have lost SOX9 expression, but the phenotypes observed likely reflect a continuum and not a binary switch.

## Methods

### Sex as a biological variable.

Our study examined male and female animals, and similar findings are reported for both sexes.

### Mice.

*Apc^fl/fl^(580S)* mice (54), *Sox9^fl/fl^* mice (55), and *Kras^LSL-G12D/+^* (56) mice have been previously described. To target *Apc* and/or *Sox9* alleles specifically in mouse colon epithelium, *CDX2P-CreER^T2^* transgenic mice (23) were first intercrossed with *Apc^fl/fl^* and/or *Sox9^fl/fl^* mice. The resulting Cre-positive *Apc^fl/+^* and/or *Sox9^fl/+^* mice were bred to *Apc^fl/fl^* mice and/or *Sox9^fl/fl^* mice to generate the following Cre-positive transgenic mice: *CDX2P-CreER^T2^ Apc^fl/fl^* (abbreviated as A)*,* *CDX2P-CreER^T2^ Sox9^fl/fl^* (abbreviated as S), *CDX2P-CreER^T2^* *Apc^fl/fl^* *Sox9^fl/fl^* (abbreviated as AS), and *CDX2P-CreER^T2^* *Apc^fl/fl^* *Sox9^fl/+^* (abbreviated as AS^het^). *CDX2P-CreER^T2^ Apc*^fl/+^ *Kras^LSL-G12D/+^* *Trp53^R270H/fl^* mice (referred to as AKP^270/fl^) have been previously described (27). Instead of using the previous mouse model that constitutively expresses *Trp53^R270H^* mutant allele under the control of the *Trp53* endogenous promoter, for this study, we used a mouse model generated by Serguei Kozlov (National Cancer Institute, NIH, Frederick, Maryland, USA) that conditionally expresses the *Trp53^R270H^* mutant allele (*Trp53^fl-R270H^*) upon Cre-mediated recombination. In *Trp53^fl-R270H^* mice, a *LoxP*-flanked cassette containing the mouse *Trp53* wild-type exons 5–11 cDNA was inserted into the intron 4 of the *Trp53^R270H^* mutant allele, which carries a modified exon 8 encoding p53R270H. This conditional *Trp53* allele allows for the expression of wild-type p53 prior to Cre-mediated recombination, after which it encodes a mouse p53R270H mutant protein. *CDX2P-CreER^T2^ Apc*^fl/+^ *Kras^LSL-G12D/+^* *Trp53^fl-R270H/fl^* mice (referred to as AKP in the present study) exhibit essentially the same tumor burden and latency as the AKP*^270/fl^* mice previously described (27). The AKP mice were further bred with *Sox9^fl/fl^* mice to generate compound mice referred to as AKP*Sox9^fl/fl^* (AKPS). All experimental compound mice were on a mixed C57BL/6 and 129 background, and littermates with similar genetic backgrounds and different genotypes were used for comparison (see breeding scheme above). All the mice were housed in specific pathogen–free conditions. After weaning, 5LOD chow (LabDiet) and automatically supplied water were provided ad libitum to mice.

### TAM treatment.

We performed i.p. injections of TAM (Sigma-Aldrich) dissolved in corn oil (Sigma-Aldrich) on adult mice aged 2–4 months. The mice received 2 daily doses of TAM, with each dose being 120 mg/kg for S, A, AS^het^, and AS mice, and 70 mg/kg for AKP and AKPS mice. The animals were euthanized upon reaching humane endpoints.

### Cell culture.

All human colon cancer cell lines were obtained from American Type Culture Collection. The mouse colon cancer cell lines harboring *Apc*, *Kras*, and *Trp53* mutant alleles (AKP1 and AKP2) were generated from colon tumors of AKP^270/fl^ mice (27). All the cell line studies are described in the Supplemental Methods.

### Establishment of colonic organoids.

Organoids were derived from proximal colon tissues of A mice (*n* = 4) or AS mice (*n* = 4) at 3–4 weeks after TAM injection. The colonic organoids were generated and propagated using a previously described Tokyo Medical and Dental University (TMDU) protocol (57) with minor modifications (58). TMDU medium lacking Wnt3a and R-spondin2 conditional media were used to select for *Apc* deletion mutation in organoids that carry *Apc^fl^* alleles. All the organoids were cultured for 4 days before being harvested for RNA analysis.

### IHC.

Mouse tissues and organoids were fixed in 10% (v/v) buffered formalin and embedded in paraffin. A human colon TMA containing 194 cases of adenocarcinoma was purchased (TissueArray.com, CO20813a); of the 194 cases, 171 samples contained tumor cells that were scored in the IHC study. IHC analysis of mouse and human tissues and organoids is detailed in the Supplemental Methods.

### Western blot analysis.

Western blot analyses of whole-cell lysates from mouse colon tissues and human or mouse colon cancer cell lines were performed as previously described (59). See the Supplemental Methods for the antibodies used.

### SOX9 promoter methylation analysis: bisulfite sequencing.

DNA methylation of the *Sox9* promoter in human colon cancer cell lines was assessed by bisulfite sequencing. See the Supplemental Methods for details.

### Gene expression.

RNA-Seq was used to profile gene expression in mouse colon tissues and organoids. Detailed information on sample description, library preparation, and data analysis can be found in the Supplemental Methods.

### Bioinformatic analysis of public data for patients with CRC.

We downloaded patient-level data for the COADREAD cohort (https://xenabrowser.net/ and http://www.cbioportal.org/), which included RNA-Seq data and clinical data for 376 patients. We also downloaded methylation (HM450) beta-values for the *SOX9* gene and *SOX9* mRNA expression values (RNA-Seq V2 RSEM) for 353 patients with CRC from TCGA (http://www.cbioportal.org/). Bioinformatic analysis is detailed in the Supplemental Methods.

### Statistics.

In the IHC study using TMA, CRC samples with SOX9 IHC scores “−” or “+/−” were designated as “0” and those with SOX9 IHC scores of 1+, 2+, or 3+ were designated as “1.” Fisher’s exact test was used to examine the association between the binary target variable Sox9_Score and other binary independent variables (grade, sex, and stage); a simple logistic regression model was used to analyze the independent variable of age. Kaplan-Meier survival curves in both mouse and human studies were compared by log-rank test. Student’s *t* tests (unpaired, 2 tailed) or Welch’s *t* tests (unpaired, 2 tailed) were used to determine the significance between experimental groups, as indicated in the figure legends. Fisher’s exact test was used to test the correlation between categorical independent variables (tumor grade and invasiveness) and SOX2 protein expression in mouse tumor models. For patient data, the Cox proportional hazard regression model was used in multivariate analyses to estimate the HRs and 95% CI. The significance of HRs was determined by the Wald test. To compare nested Cox models, a likelihood ratio test was performed to evaluate whether interaction terms significantly improved model fit. A χ^2^ test or Fisher’s exact test was used wherever appropriate to test the correlation between categorical independent variables (e.g., sex, local invasion depth, lymph node involvement, tumor site) and *SOX9* gene expression; a paired *t* test (2-tailed) was used for testing the association of the independent continuous variable (age at diagnosis) with *SOX9* gene expression. Spearman’s correlation was used to assess the relationship between DNA methylation and *SOX9* gene expression. A *P* value less than 0.05 was considered statistically significant for all the analyses (with appropriate multiplicity adjustments where needed).

### Study approval.

Animal husbandry and experimental procedures were carried out under approval from the University of Michigan’s IACUC (PRO00010785 and PRO00012388) and according to Michigan state and US federal regulations.

### Data availability.

Data values for all data presented in graphical form or presented as means can be found in the Supporting Data Values file. The RNA-Seq data are available in the NCBI’s Gene Expression Omnibus (GEO) database under accession number GSE239716. Materials will be made available upon request.

## Author contributions

YF conceived and carried out experiments, performed data analyses, and drafted the manuscript. NZ helped with animal studies. KB performed RNA-Seq data analysis. JL and QL conducted bioinformatics analysis on patient data obtained from TCGA. CP conducted statistical analysis for the IHC study on human TMA samples. MG helped with animal studies. NA and YZ conducted histopathological and IHC evaluation on human colon tumor TMA samples. VB supervised bioinformatics and statistical analyses. JRS provided *Sox9^fl^* mice and scientific guidance about their use. KRC provided feedback on designing and performing experiments. ERF conceived and supervised the study and wrote the manuscript. All the authors reviewed the manuscript and had final approval of the submitted version.

## Supplementary Material

Supplemental data

Unedited blot and gel images

Supporting data values

## Figures and Tables

**Table 1:**
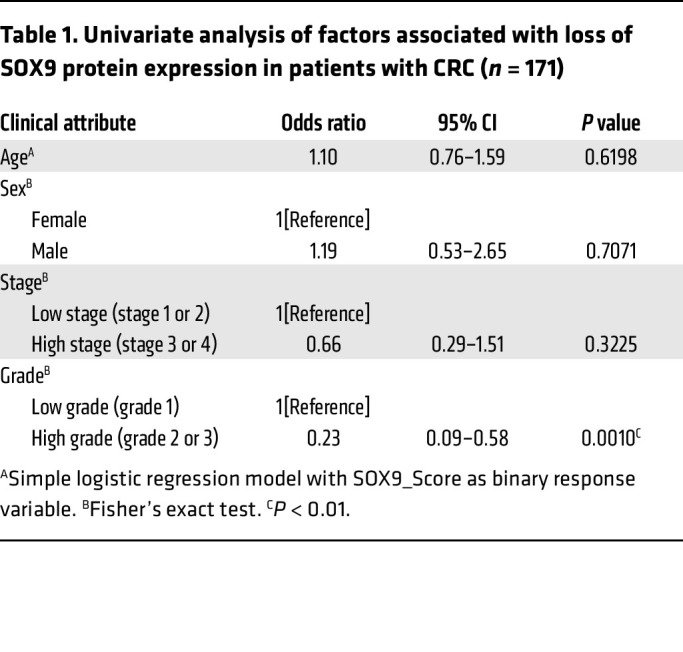
Univariate analysis of factors associated with loss of SOX9 protein expression in patients with CRC (*n* = 171)

**Table 3:**
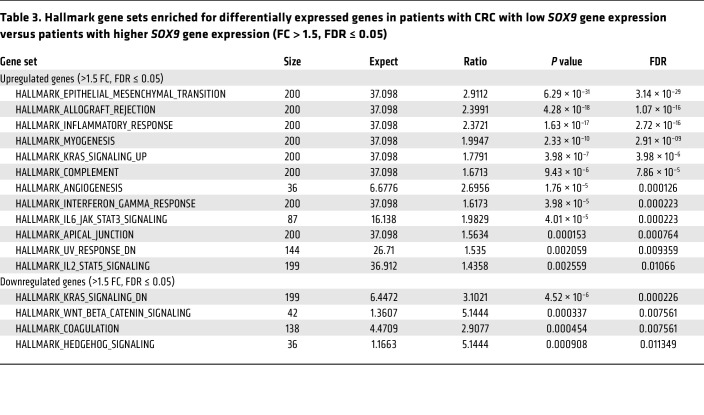
Hallmark gene sets enriched for differentially expressed genes in patients with CRC with low *SOX9* gene expression versus patients with higher *SOX9* gene expression (FC > 1.5, FDR ≤ 0.05)

**Table 2:**
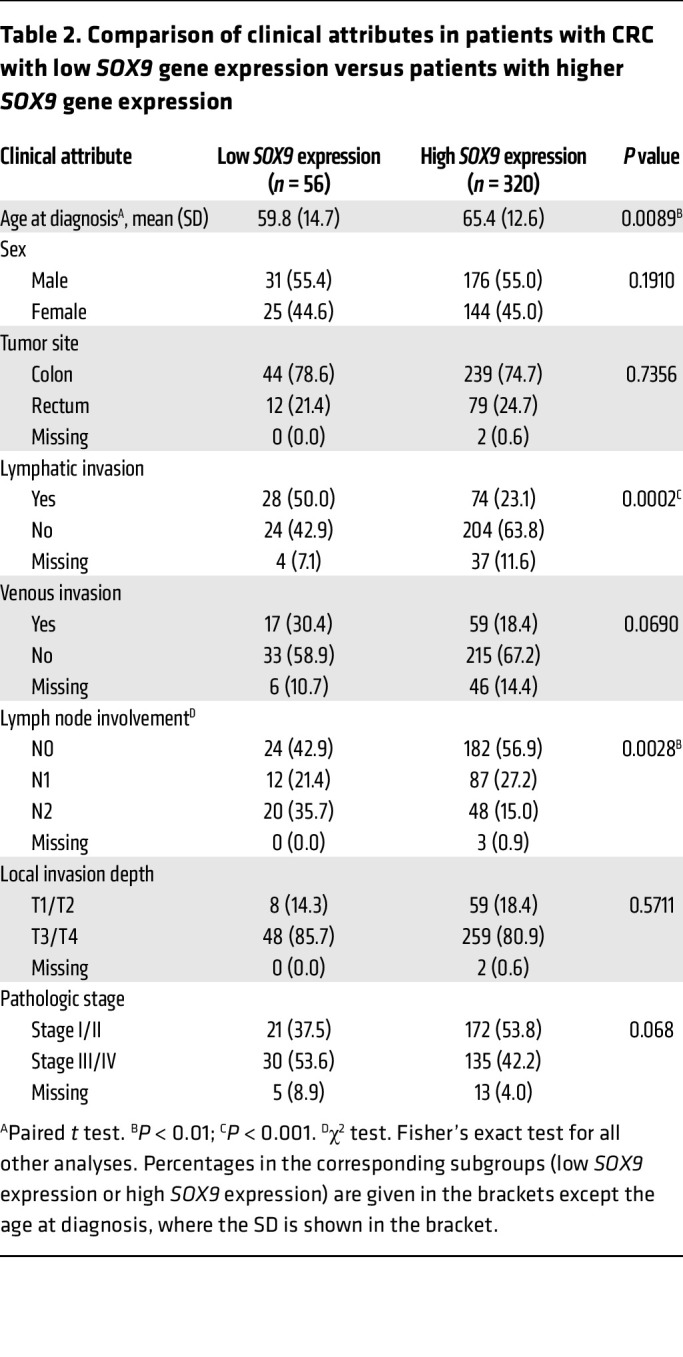
Comparison of clinical attributes in patients with CRC with low *SOX9* gene expression versus patients with higher *SOX9* gene expression

## Version 1 04/03/2025

In-Press Preview

## Version 2 06/02/2025

Electronic publication
