# Supplementary material for: SOX9 suppresses colon cancer via inhibiting epithelial-mesenchymal transition and SOX2 induction
Source: J Clin Invest. 2025 Apr 3;135(11):e184115. doi: 10.1172/JCI184115 (PMC12126244; doi:10.1172/JCI184115)
Supplement: Supplemental data [file jci-135-184115-s237.pdf]

Supplemental Materials

SOX9 suppresses colon cancer via inhibiting epithelial-mesenchymal transition and SOX2 induction

Ying Feng,[1] Ningxin Zhu,[1,] Karan Bedi,[2] Jinju Li,[2] Chamila Perera,[2] Maranne Green,[1] Naziheh Assarzadegan,[3] Yali Zhai,[3] Qingzhi Liu,[2] Veerabhadran Baladandayuthapani,[2,4,5] Jason R Spence,[1,5,6] Kathleen R. Cho,[3,5] Eric R. Fearon[1,3,5,7]*

[1]Department of Internal Medicine, University of Michigan Medical School, Ann Arbor, Michigan, USA. [2]Department of Biostatistics, University of Michigan School of Public Health, Ann Arbor, Michigan, USA. [3]Department of Pathology, [4]Department of Computational Medicine and Bioinformatics, [5]the Rogel Cancer Center, [6]Department of Cell and Developmental Biology, and [7]Department of Human Genetics, University of Michigan Medical School, Ann Arbor, Michigan, USA.

*Current Address – *Department of Infectious Disease, the First Affiliated Hospital, Zhejiang University School of Medicine, Hangzhou, China*

Running Head: SOX9 tumor suppressor role in colorectal cancer

Keywords: Colorectal cancer, SOX9, tumor suppressor, Wnt pathway, adenomatous polyposis coli, beta-catenin, epithelial mesenchymal transition, SOX2

This PDF includes:
Supplemental methods
Supplemental Figures 1-17
Supplemental Tables 1-6

Supplemental methods

Cell culture

Human colon cancer cell lines HT-29, SW480, HCT116, and DLD-1 cells were grown in Dulbecco's minimal essential medium (Thermo Fisher Scientific) supplemented with 10% fetal bovine serum. RKO was cultured in minimum essential medium (Thermo Fisher Scientific) supplemented with 10% fetal bovine serum. For treatment with the demethylating agent and the histone deacetylase inhibitor, RKO cells were first treated with vehicle (DMSO) or treated with 1 or 5 μM 5-Aza-2′-deoxycytidine (5-AzaD) (Sigma-Aldrich) for 3 days to induce DNA demethylation. During the third day of treatment with vehicle or 5-AzaD, the cells were further incubated with vehicle or 0.5 μM trichostatin A (TSA) (Sigma-Aldrich) for 24 h to inhibit histone deacetylases. Protein lysates were then collected from these cells and subject to Western blot analysis for assessment of SOX9 expression. The mouse colon cancer cell lines AKP1 and AKP2 were grown in Advanced DMEM/F12 medium supplemented with 10% fetal bovine serum, L-glutamine, and penicillin/streptomycin. To generate AKP cells stably overexpressing SOX2 or an empty control vector, AKP1 and AKP2 cells were infected with a pLVX-IRES-Puro lentiviral vector (Clontech Laboratories) carrying a cDNA for human SOX2 or the empty vector, respectively. The infected AKP cells were selected with 1.25 μg/mL of puromycin (Sigma-Aldrich) for two generations, after which the proteins were subjected to Western blot analysis.

Immunohistochemistry (IHC)

Sections of the paraffin-embedded mouse and human tissues and organoids were subjected to IHC analysis, as previously described (1). The following primary antibodies were used for IHC analysis: mouse anti–β-catenin (1:500; #610154, BD Biosciences), rabbit anti-SOX9 (1:500 for mouse tissues; ab185966, Abcam), rabbit anti-SOX9 (1:500 for human TMA; AB5535, MilliporeSigma), goat anti–E-cadherin (1:500; AF748, R&D Systems), rabbit anti-vimentin

(1:500; #2707-1, Epitomics), mouse anti-BrdU (1:500; #555627, BD Biosciences), rabbit anti-SOX2 (1:150; #14962, Cell Signaling Technology), rabbit-anti-lysozyme (1:1500; #A0099, Dako), and rabbit-anti-villin (1:200; GTX109940, GeneTex) . Histochemical identification of intestinal goblet cells was performed on paraffin sections with Alcian blue (Sigma-Aldrich) followed by H&E staining. SOX9 immunostaining on human TMA samples was scored based on the percentage of positive cells and intensity in the cell nucleus: "−", absent in all cells; "+/−", <10% positive cells with minimal staining; "1+", 10-25% positive cells with moderate staining; "2+", 26-50% positive cells with strong staining; "3+", >50% positive cells with very strong staining. Staining was scored by two independent viewers (YF, NA), and rare discordant scores were resolved by consensus review. Human CRC samples on TMA with no or negligible SOX9 staining (score "-" or "+/−") were considered to have a loss of SOX9 protein expression. Representative examples of staining are shown in Figure 6.

Western Blot Analysis

Western blot analyses of whole-cell lysates from mouse colon tissues and human or mouse colon cancer cell lines were performed as previously described (2). The following antibodies were used: rabbit anti-SOX9 (1:5000; AB5535, MilliporeSigma**)**, mouse anti-active β-catenin (1:2000; #05-665, MilliporeSigma), mouse anti–p-ERK (1:2000; sc-7383, Santa Cruz Biotechnology, Santa Cruz, CA), rabbit anti-ERK1/2 (1:2000; #9102, Cell Signaling Technology, Danvers, MA), rabbit anti-SOX2 (1:2000; #14962, Cell Signaling Technology), mouse anti-E-cadherin (1:10000; # 562869, BD Biosciences), rat anti-N-cadherin (1:1000; MNCD2, Developmental Studies Hybridoma Bank), rabbit anti-vimentin (1:5000; #2707-1, Epitomics), rabbit anti-Slug (1:1000; #9585, Cell Signaling Technology), rabbit anti-TWIST1 (1:1000; #90445, Cell Signaling Technology) and mouse anti–β-actin (1:10000; A1978, MilliporeSigma).

***SOX9* promoter methylation analysis: bisulfite sequencing**

Genomic DNA was extracted from 2 human CRC cell lines, RKO and HT29, and was bisulfite treated with EZ DNA Methylation-Gold kit (Zymo Research) per the manufacturer's instructions. The bisulfite-treated DNA was amplified using PCR primers encompassing the CpG islands in the SOX9 promoter and first exon. The amplified DNA fragments were cloned into the pCR2.1 vector using the TOPO-TA cloning kit (Invitrogen). At least 5 clones were then randomly selected and sequenced for each region of interest. The lollipop diagrams showing the methylation status for each cell lines were generated using BiQ Analyzer software (3). Primers used for *SOX9* bisulfite PCR were: F1 (forward), 5′- GGGGTTGGAGAATGATTTGTTAGAG-3′ and R1 (reverse), 5′- TCTAAACTAAAATCTACCCCCACCTC-3′; F2 (forward), 5′-GGGTAGTTGTGAATTGGTTATTT-3′ and R2 (reverse), 5′-CATAAAAAAATCCAAAAAATTCATAC-3′; F3 (forward), 5′- TTTTGGATTTTTTTATGAAGATGAT-3′ and R3 (reverse), 5′- TAACCTTTAAACACCTAACTAACC-3′.

Genotyping of the *Apc* and *Sox9* gene loci

DNAs were extracted from the proximal colon mucosa of 3 A mice, 3 AS^{het} mice, 3 AS mice, and 3 Cre-negative littermate controls, all sacrificed at 29–35 days after receiving two daily doses of TAM. The presence of the *Apc* and *Sox9* wild-type alleles, flox alleles, and recombined mutant alleles (flox-delete) was detected by PCR with the DNAs above. The following primers were used: *Sox9* (flox forward), 5′- CCGGCTGCTGGGAAAGTATATG -3′, *Sox9* (flox-delete forward), 5′- CTCCGGTAGCAAAGGCGTTTAG -3′, and *Sox9* (reverse), 5′-CGCTGGTATTCAGGGAGGTACA-3′; *Apc* (flox forward), 5′-GTTCTGTATCATGGAAAGATAGGTGGTC -3′, *Apc* (flox reverse), 5′-GAGTACGGGGTCTCTGTCTCAGTGAA -3′, and *Apc* (flox-delete reverse), 5′-CACTCAAAACGCTTTTGAGGGTTGATTC-3′.

Gene expression

RNA sequencing (RNA-seq) was used to profile gene expression in mouse colon tissues from the following mice: (1) control mice (littermates without Cre or *Apc/Sox9* floxed alleles); (2) *CDX2P-CreER^{T2} Sox9$^{flox/flox}$* (abbreviated as S) mice; (3) *CDX2P-CreER^{T2} Apc$^{flox/flox}$* (abbreviated as A) mice; (4) *CDX2P-CreER^{T2} Apc$^{flox/flox}$ Sox9$^{flox/+}$* (abbreviated as AS^{het}); (5) *CDX2P-CreER^{T2} Apc$^{flox/flox}$ Sox9$^{flox/flox}$* (abbreviated as AS) mice. Six mice per group except seven mice for AS^{het} were treated with tamoxifen (TAM) to induce deletions in *Apc* and *Sox9* in the colon, cecum, and ileum. Total RNAs were collected from proximal colon tissues of mutant and control mice above after 30-40 days post TAM treatment. We also collected total RNAs from organoids that were derived from mouse colon of A and AS mice following TAM-induced gene inactivation (4 mice per group). RNA was extracted using Trizol (Invitrogen) and purified with miRNeasy Mini Kit (Qiagen). RNA sequencing was performed by the University of Michigan Advanced Genomics Core, with PolyA+, stranded libraries constructed and subsequently subjected to 151 paired-end cycles on the NovaSeq-6000 platform (Illumina). Initial data analysis was performed by the University of Michigan Bioinformatics Core. Raw reads were trimmed using Cutadapt (v2.3). FastQC (v0.11.8) was used to ensure the quality of data. Reads were mapped to the reference genome GRCm38 (ENSEMBL) or mm10 (Gencode vM21) using STAR (v2.6.1b) (4) and gene count estimates were assigned using RSEM (v1.3.1) (5). Alignment options followed ENCODE standards for RNA-seq (https://github.com/alexdobin/STAR/blob/master/doc/STARmanual.pdf). FastQC was used in an additional post-alignment step to ensure that only high-quality data were used for expression quantitation and differential expression.

Differential gene expression across groups was analyzed with DESeq2 in R. For enrichment test, we mapped mouse genes to human homologs using only 1-to-1 best homologs from NCBI Homologene build 68. The obtained differentially expressed genes (DEGs, defined as fold change (FC) >1.5 and FDR adjusted *P* value <= 0.05) were used for gene set enrichment

analysis with a web-based functional enrichment analysis tool called WebGestalt (WEB-based GEne SeT AnaLysis Toolkit) available at http://www.webgestalt.org/. A similar approach was also employed to compare gene expression in human CRCs.

Gene counts produced by RSEM and the raw sequencing data are available in the NCBI's Gene Expression Omnibus (GEO) database under the accession number GSE239716.

The RNAs from mouse colon tissues and organoids used for RNA-seq were also subject to quantitative reverse transcription (RT)-PCR (qRT-PCR) to measure the *Sox9* gene expression. The qRT-PCR analysis was performed as previously described (6) and the following primers were used: *Sox9* (forward), 5′- GACTCCCCACATTCCTCCT-3′ and *Sox9* (reverse), 5′-CAGCTTGCACGTCGGTTT-3′; *Actb* (forward), 5′- GCCTTCCTTCTTGGGTATGG-3′ and *Actb* (reverse), 5′- GCCTGGGTACATGGTGGT -3′.

Bioinformatic analysis of public data for human CRC patients

Patient-level data for TCGA COADREAD cohort was downloaded from https://xenabrowser.net/ and http://www.cbioportal.org/, which include RNA-seq data and clinical data for 376 patients. R 4.1.0 was used to carry out the computations for all the analyses. For survival analyses of overall survival (OS), disease-specific survival (DSS), and progression-free interval (PFI), we employed multivariate Cox proportional hazards models, including *SOX9* gene expression (log_2-transformed), age at diagnosis, sex, local invasion depth, lymph node involvement, and tumor site as covariates. The time of diagnosis was used as the baseline when defining OS, DSS, and PFI. The effect of MSI status on the association between *SOX9* expression and survival was investigated in two ways: (1) by applying the multivariate Cox proportional hazards models to the subgroup of non-MSI-H patients and comparing these results with the entire cohort; and (2) by conducting likelihood ratio test to determine whether adding an interaction term between MSI

status and *SOX9* expression significantly improved model fit in nested models. To identify a prognostically meaningful cutoff for *SOX9* gene expression and further examine the relationship of *SOX9* expression with various clinicopathological factors and other gene expressions, we used the surv_cutpoint() function from the “survminer” R package. This function selects the optimal cutoff for a continuous variable based on the maximally selected log-rank statistic, thereby identifying the threshold for *SOX9* expression that yields the greatest differences in survival probabilities between the low- and high-expression groups. Gene mutation profiles for the same cohort of CRC patients were downloaded from https://xenabrowser.net/ and the non-silent somatic mutations found in selective cancer-related genes were counted and compared for *SOX9* low and high groups. Differential gene expression between the patient groups with low and high *SOX9* gene expression, as well as gene set enrichment analysis for human CRCs, were conducted following the same approach as described for the mouse study.

We downloaded methylation (HM450) beta-values for the *SOX9* gene (for genes with multiple methylation probes, we selected the probe most anti-correlated with expression) and *SOX9* mRNA expression values (RNA-Seq V2 RSEM) for 353 CRC patients from TCGA via http://www.cbioportal.org/. Spearman correlation was used to assess the relationship between DNA methylation and *SOX9* gene expression.

Supplemental reference

1. Feng Y, et al. Tissue-specific effects of reduced beta-catenin expression on adenomatous polyposis coli mutation-instigated tumorigenesis in mouse colon and ovarian epithelium. *PLoS Genet.* 2015;11(11):e1005638.
2. Kolligs FT, et al. Neoplastic transformation of RK3E by mutant beta-catenin requires deregulation of Tcf/Lef transcription but not activation of c-myc expression. *Mol Cell Biol.* 1999;19(8):5696-5706.
3. Bock C, et al. BiQ Analyzer: visualization and quality control for DNA methylation data from bisulfite sequencing. *Bioinformatics.* 2005;21(21):4067-4068.
4. Dobin A, et al. STAR: ultrafast universal RNA-seq aligner. *Bioinformatics.* 2013;29(1):15-21.
5. Li B, and Dewey CN. RSEM: accurate transcript quantification from RNA-Seq data with or without a reference genome. *Bmc Bioinformatics.* 2011;12:323.
6. Sakamoto N, et al. BRAF(V600E) cooperates with CDX2 inactivation to promote serrated colorectal tumorigenesis. *Elife.* 2017;6:e20331.

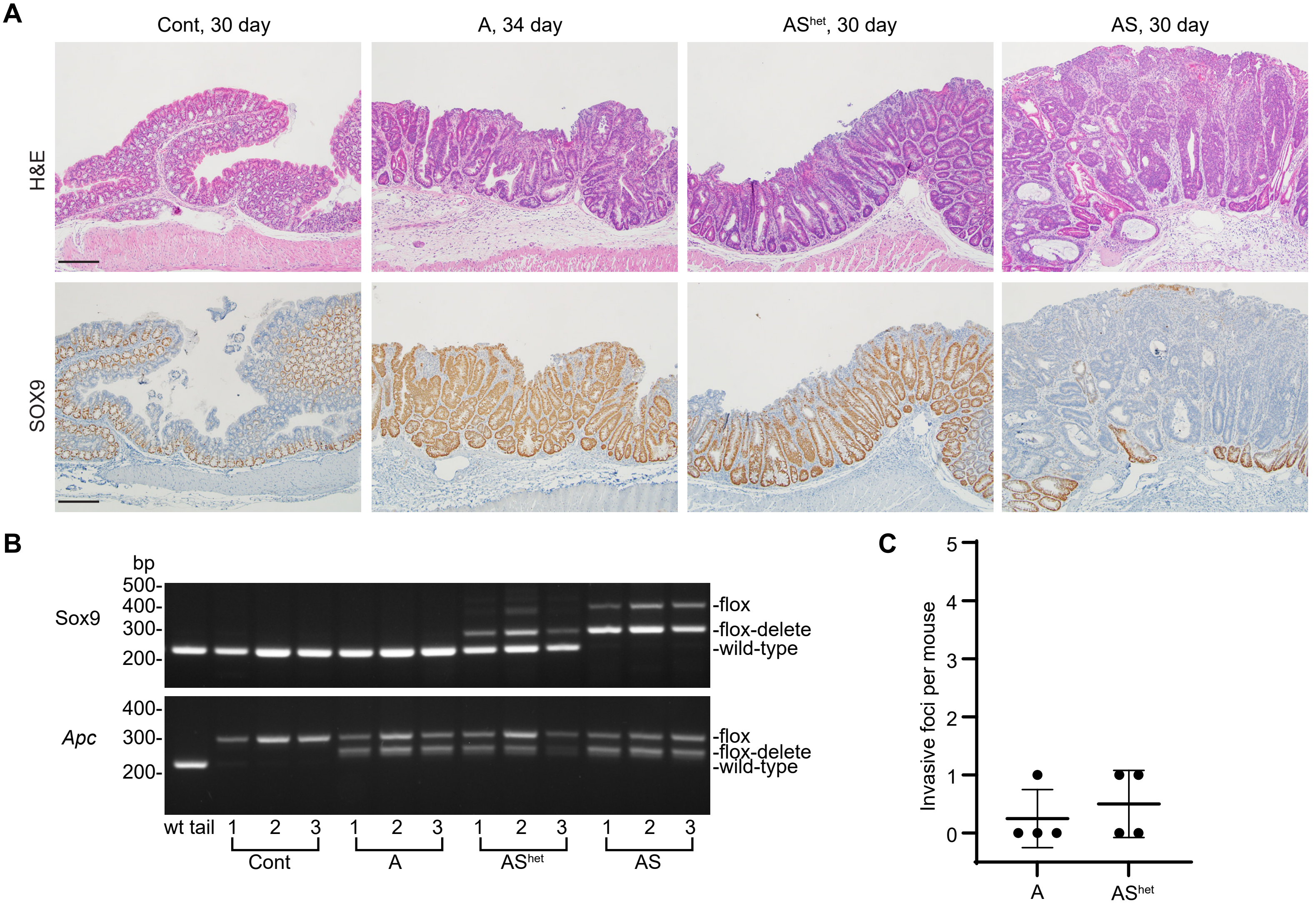

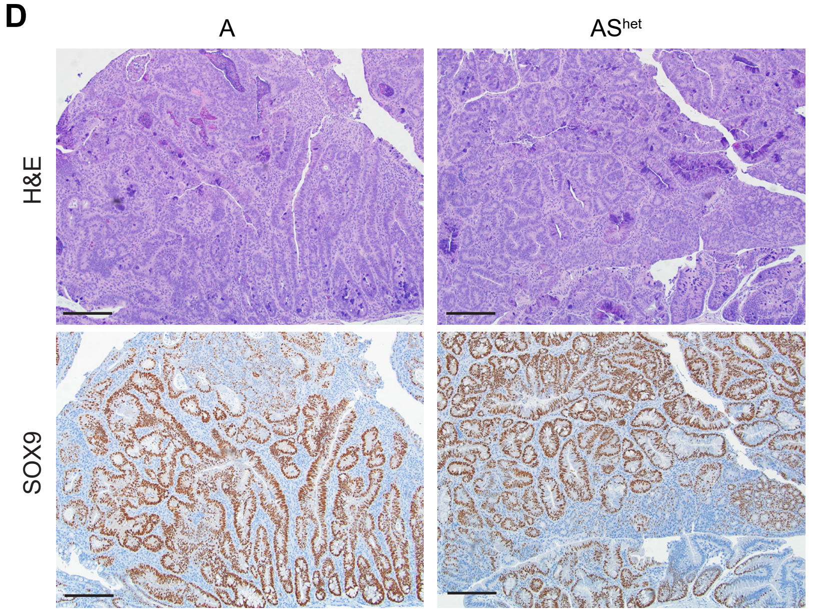

Supplemental Figure 1. Mouse colon lesions with inactivation of two *Apc* alleles and one *Sox9* allele (AS^{het}) showed similar features to lesions with *Apc* inactivation (A) only. (**A**) H&E staining (top panels) and IHC staining for SOX9 (bottom panels) of proximal colon tissues from an A mouse, an AS^{het} mouse, an AS mouse, and a Cre negative littermate control at 30 - 34 days after 2 daily doses of TAM. Scale bars, 200 μm. (**B**) Genotyping of the *Apc* and *Sox9* loci was performed using DNA extracted from the proximal colon mucosa of 3 A mice, 3 AS^{het} mice, 3 AS mice, and 3 Cre-negative littermate controls, all sacrificed at 29–35 days after receiving two daily doses of TAM. The presence of the *Apc* and *Sox9* wild-type alleles, flox alleles, and recombined mutant alleles (flox-delete) was detected by PCR. (**C**) Quantification of invasive foci per mouse in colon, cecum, and ileum tissues. Tissue sections were prepared from the surgical areas as described in Figure 2D from A mice (n = 4) and AS^{het} mice (n = 4) at 70-134 days after 2 daily doses of TAM, and invasive foci were counted for all 6 surgical areas from each mouse (one tissue section per surgical area). P = 0.537 in Student's t test with comparison of AS^{het} mice versus A mice, and error bar denotes SD. (**D**) H&E staining (top panels) and IHC staining for SOX9 (bottom panels) of proximal colon tissues from an A mouse and an AS^{het} mouse, taken at 77 days and 81 days, respectively, after two daily doses of TAM. Scale bars, 200 μm.

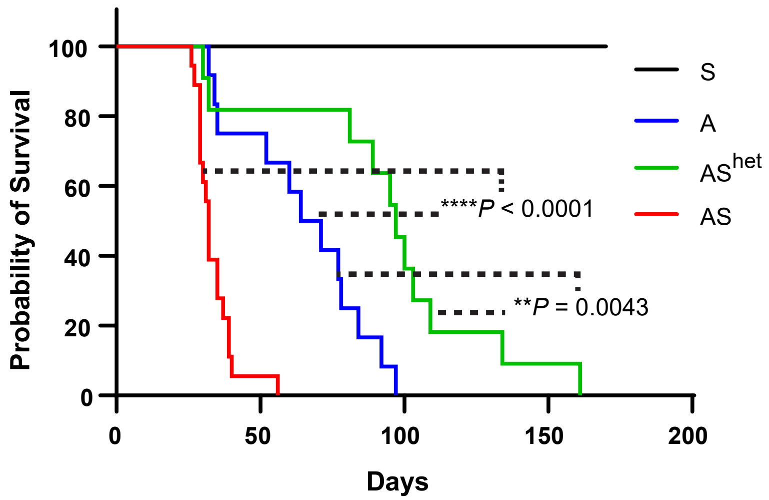

Supplemental Figure 2. Mice with combined inactivation of *Apc* and *Sox9* (AS) in colon epithelium have reduced survival compared to mice with *Apc* inactivation (A) only. Kaplan-Meier survival curves of S (black; *n* = 5), A (blue; *n* = 12), AS^{het} (inactivation of two *Apc* alleles and one *Sox9* allele, green; *n* = 11), and AS (red; *n* = 18) mice, following two daily doses of TAM (120 mg/kg). ****$P < 0.0001$ when comparing AS mice (median survival = 32 days) to A mice (median survival = 67.5 days) using log-rank (Mantel-Cox) test; **$P = 0.0043$ when comparing AS^{het} mice (median survival = 97 days) to A mice.

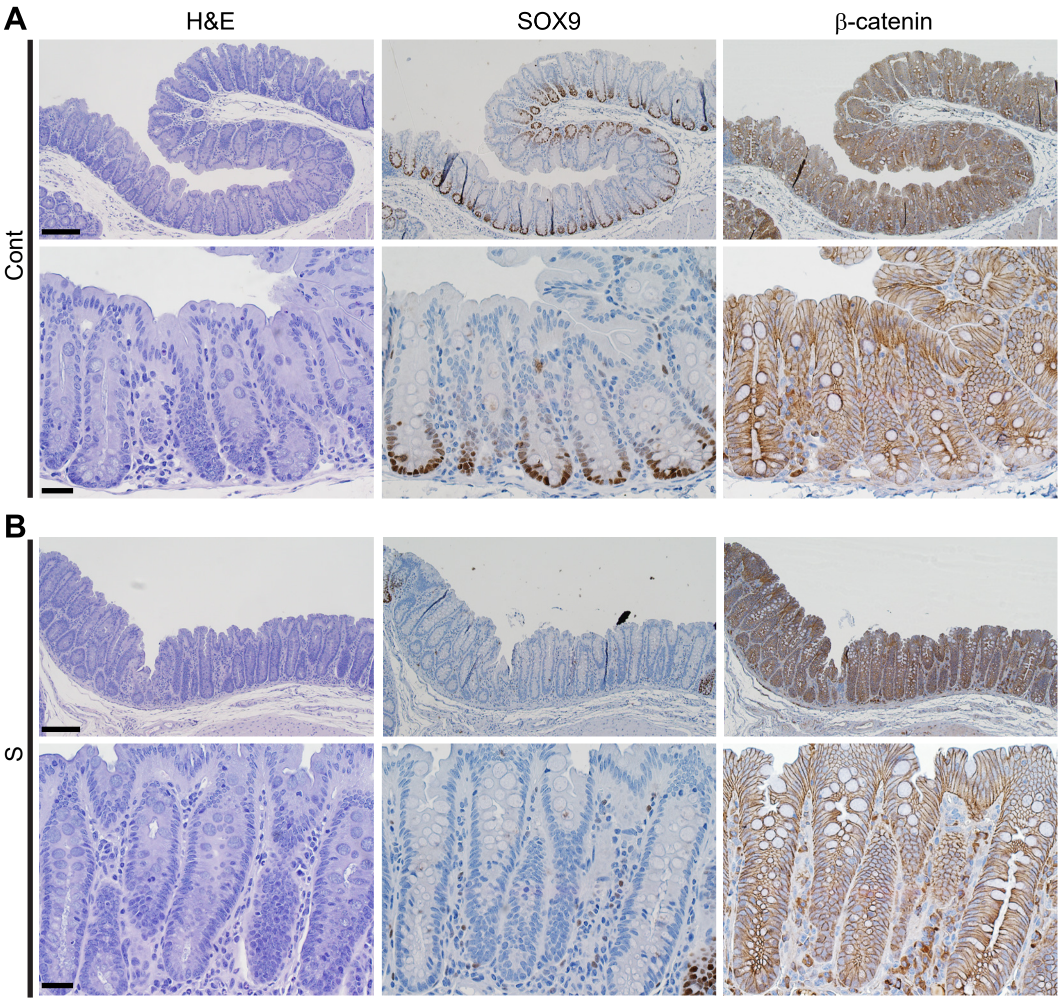

Supplemental Figure 3. Expression of SOX9 and β-catenin in mouse colon epithelium with bi-allelic *Sox9* inactivation (S). Representative photomicrographs of H&E staining and IHC staining for SOX9 and β-catenin in proximal colon tissues of a Cre negative control mouse (**A**) and a S mouse at 125 days after two daily doses of TAM (**B**). Scale bars, 100 μm for low magnification images (top panels); 20 μm for high magnification images (bottom panels).

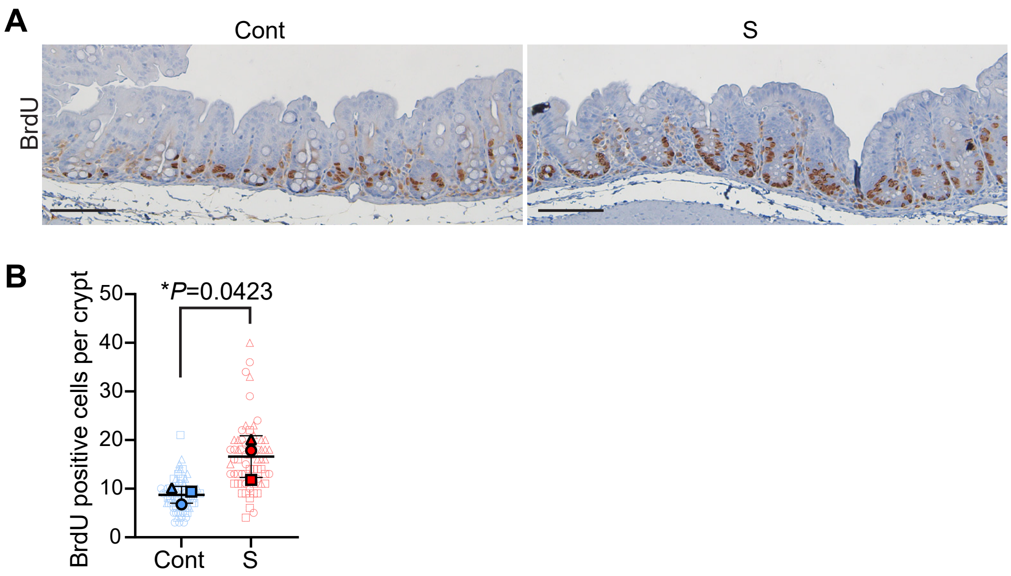

Supplemental Figure 4. *Sox9* inactivation alone (S) in mouse colon epithelium leads to increased proliferation. (**A**) IHC staining for BrdU in proximal colon of a S mouse and a Cre-negative control mouse (Cont) at 35 days after two daily doses of TAM. Scale bars, 100 μm. (**B**) Quantification of proliferating cells per crypt, as assessed by BrdU incorporation, from S mice (*n* = 3, total 73 crypts) and Cont mice (*n* = 3, total 65 crypts) at 35 days post TAM. Solid shapes represent the mean number of BrdU-positive cells per crypt for each mouse; smaller shapes represent individual values per crypt (20–27 crypts/mouse). *P = .0423 (Student's t test, S vs. Cont).

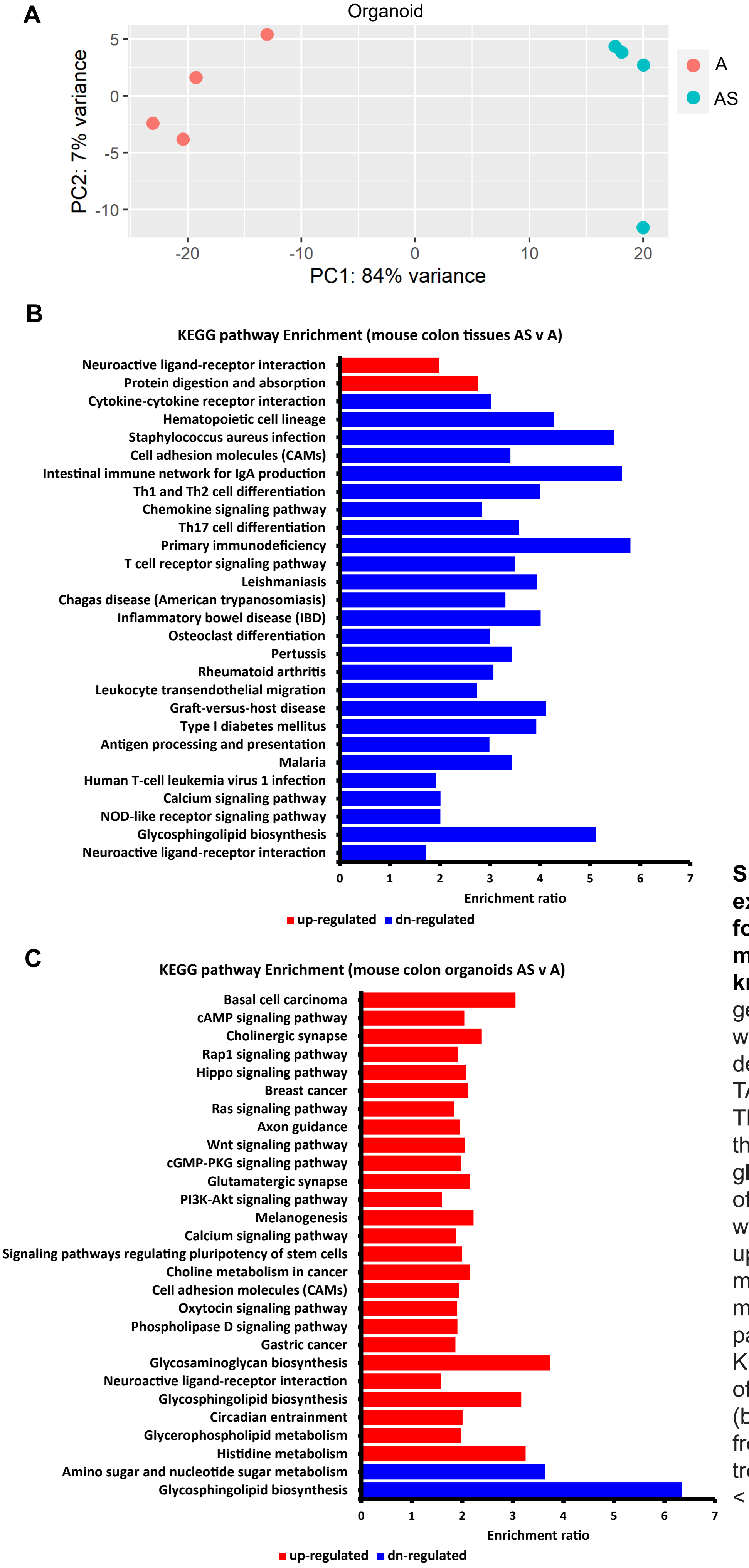

Supplemental Figure 5. Differential gene expression and KEGG pathway analysis for genes up- and down-regulated in mouse colon cells with conditional knockout of *Apc* and *Sox9.* (**A**) Global gene expression analyses were performed with RNAs from mouse colon organoids derived from A mice, and AS mice following TAM treatment (n = 4 for each genotype). The principal components analysis showed the AS mutant organoids clearly had distinct global patterns of gene expression from that of the A mutant organoids. (**B**) KEGG pathways overrepresented in the lists of genes up- (red) and down-regulated (blue) in mouse colon tissues from AS mice versus A mice following TAM treatment. The enriched pathways with FDR < 0.05 are shown. (**C**) KEGG pathways overrepresented in the lists of genes up- (red) and down-regulated (blue) in mouse colon organoids derived from AS mice versus A mice following TAM treatment. The enriched pathways with FDR < 0.05 are shown.

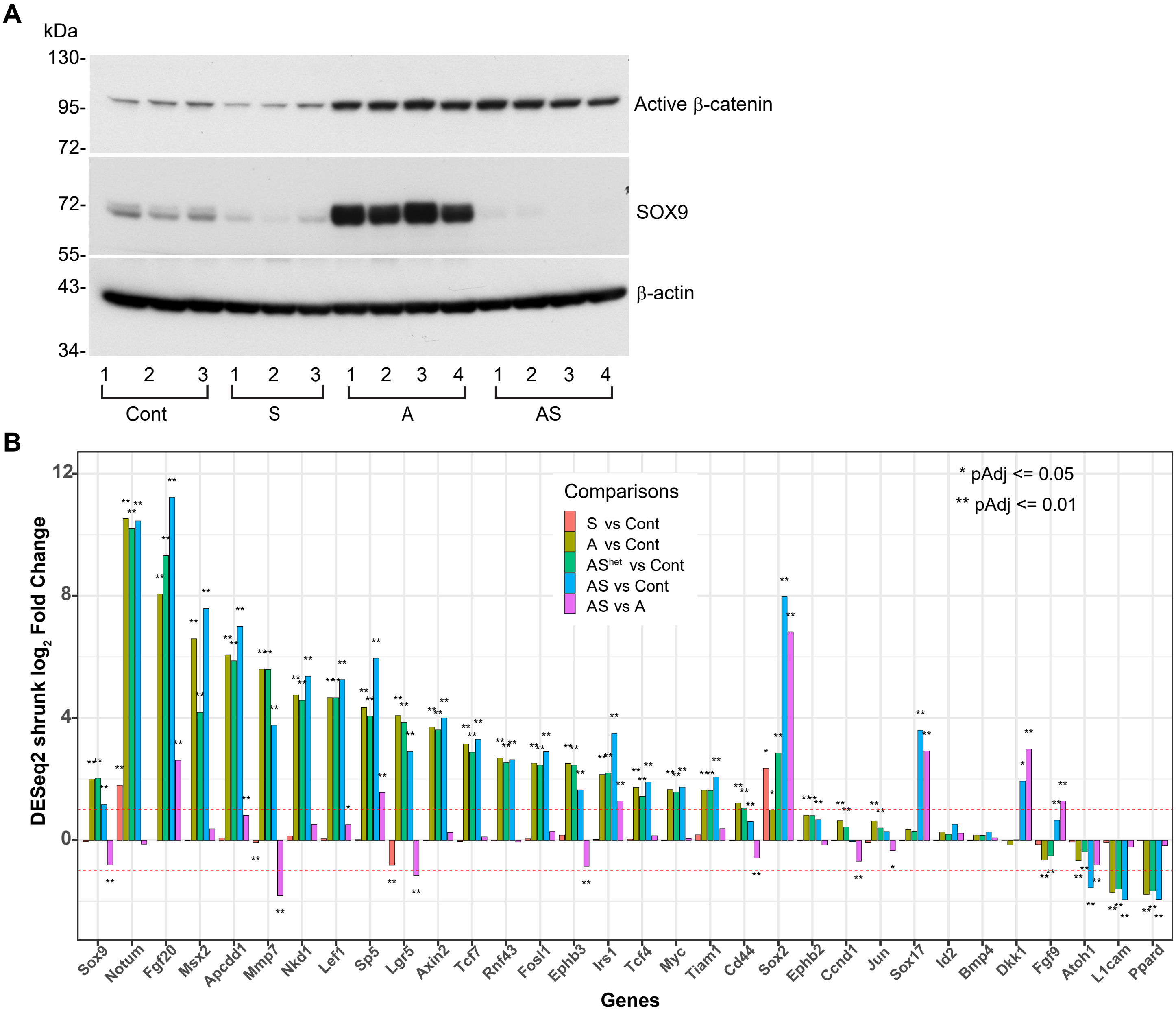

Supplemental Figure 6. Wnt signaling pathway in mouse colon epithelium with *Apc* and/or *Sox9* inactivation. (**A**) Western blot analysis for active β-catenin and SOX9 in proximal colon tissues of S (*n* = 3), A (*n* = 4), AS (*n* = 4), and control mice (*n* = 3) following TAM-induced gene targeting. The level of β-actin serves as a loading and transfer control. (**B**) Expression of *Sox9* and 31 other known canonical Wnt target genes were examined in RNA-seq data generated from the proximal colon tissues of S (*n* = 6), A (*n* = 6), AS^{het} (*n* = 7), AS (*n* = 6), and control mice (*n* = 6) following TAM-induced gene targeting. Gene expression is shown as a shrunken $\log_2$ fold change estimated by DESeq2 in the following comparisons: S vs Cont, A vs Cont, AS^{het} vs Cont, AS vs Cont, and AS vs A. One *asterisk* denotes pAdj <= 0.05; two *asterisks* denote pAdj <= 0.01. Dotted line indicates $\log_2$ fold change threshold of 1.0.

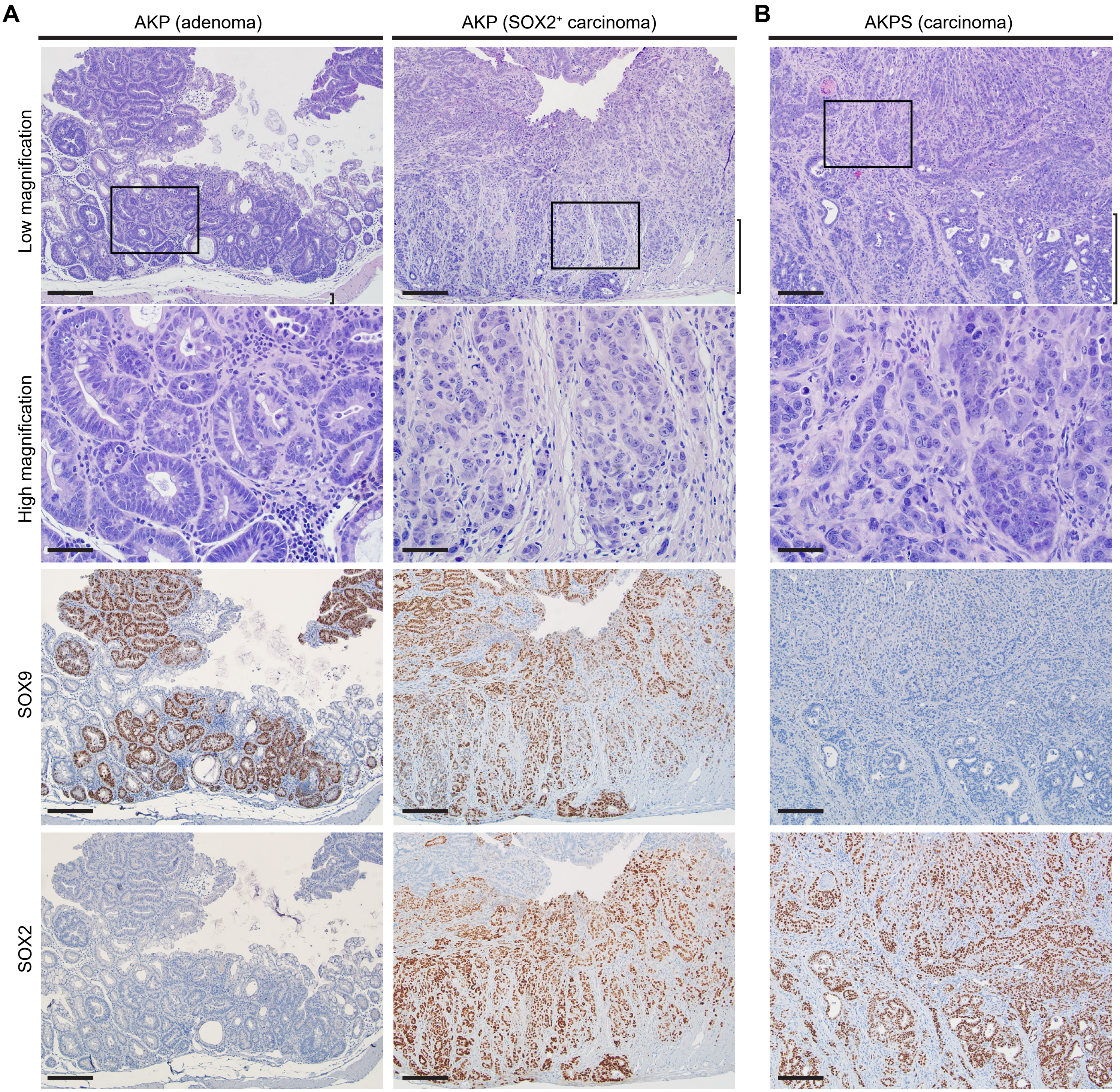

Supplemental Figure 7. SOX2 expression is associated with elevated tumor grade and increased invasiveness in a mouse model of CRC, while *Sox9* deletion results in a higher incidence of tumors exhibiting elevated SOX2 expression. H&E stains (top two rows of panels) and immunohistochemical stains for SOX9 and SOX2 (bottom two rows of panels) are shown for an adenoma and an invasive colon tumor from an AKP mouse (**A**) and an invasive colon tumor from an AKPS mouse (**B**). Tumors were collected from the proximal colon after 3-4 months following TAM induction. The representative photomicrographs of H&E-stained sections are displayed with low-power magnification in the top panels, and the boxed areas are shown at high-power magnification in the panels directly below. The brackets indicate the muscular layer. Scale bars: 200 μm for low magnification images (top 1st, 3rd, and 4th rows of panels); 50 μm for high magnification images (2nd row of panels).

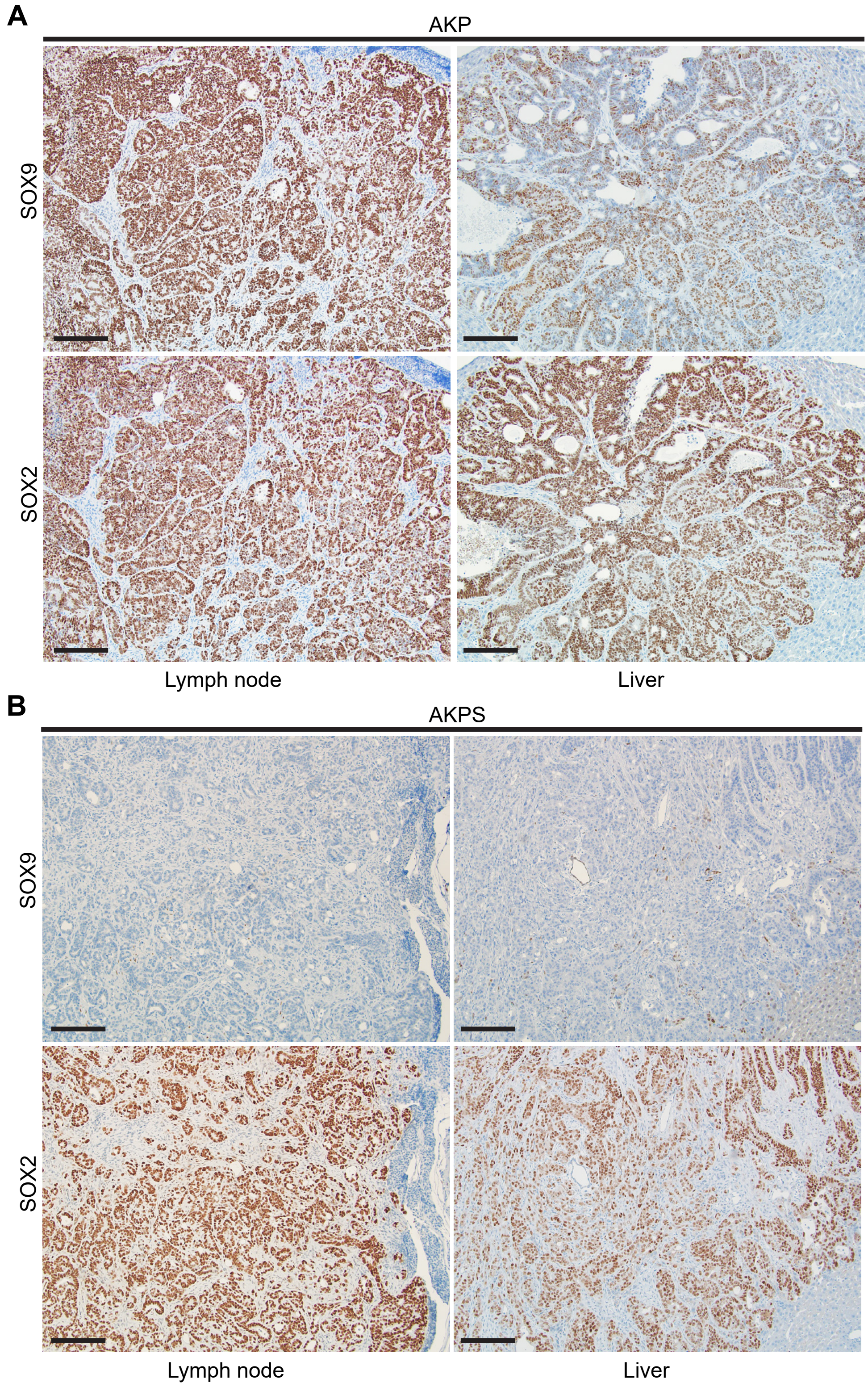

Supplemental Figure 8. SOX2 expression correlates with increased metastasis in a mouse CRC model, while *Sox9* deletion results in a higher incidence of tumors exhibiting elevated SOX2 expression. Representative photomicrographs of immunohistochemical stains for SOX9 (top panels) and SOX2 (bottom panels) are shown for the lymph node and liver metastases found in an AKP mouse (**A**) and an AKPS mouse (**B**). Tumors were collected after 3-4 months following TAM induction. Scale bars: 200 μm.

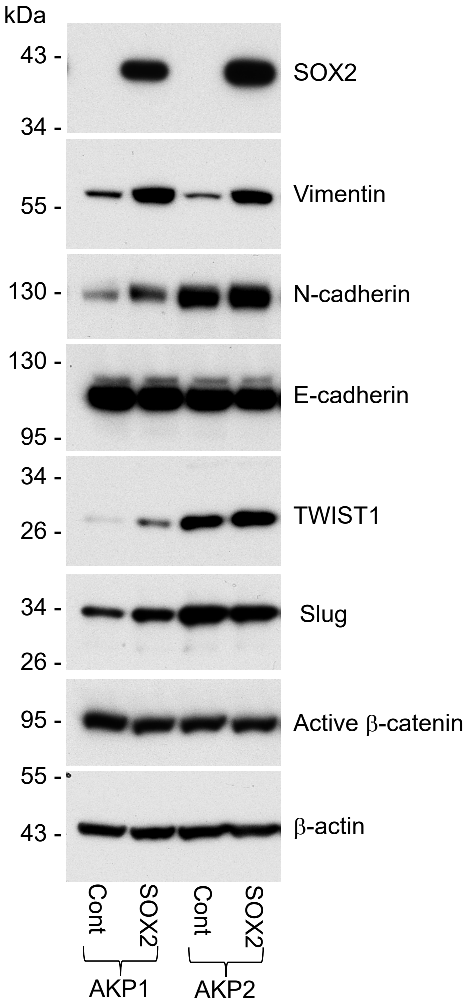

Supplemental Figure 9. Increased expression of EMT markers and EMT-inducing transcription factors in mouse AKP colon cancer cell lines with ectopic expression of SOX2. Western blot analysis of SOX2, vimentin, N-cadherin, E-cadherin, TWIST1, Slug, and active β-catenin in mouse colon cancer cell lines, AKP1 and AKP2, stably overexpressing SOX2 or a control empty vector (Cont). β-actin was used as a loading and transfer control.

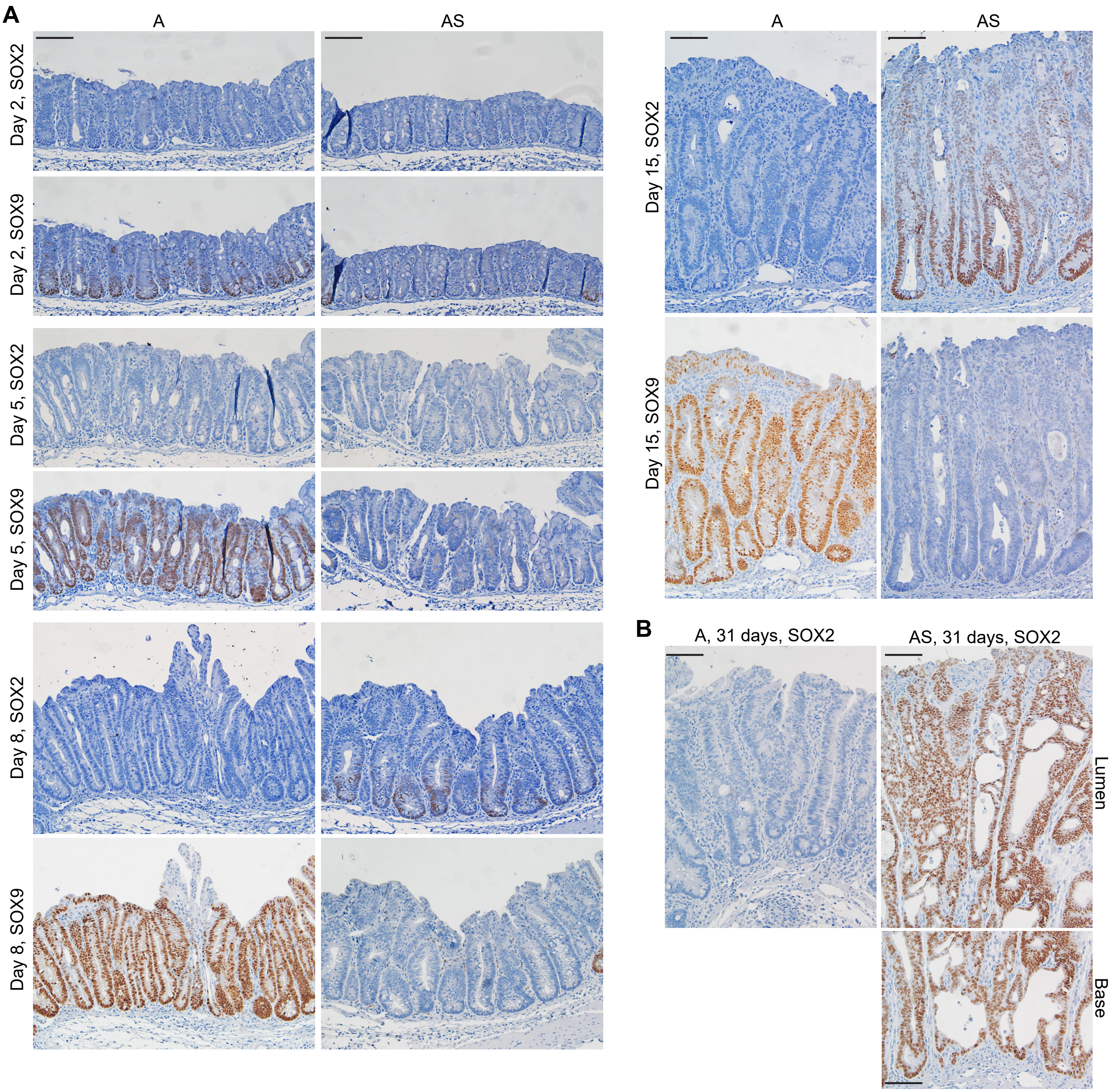

Supplemental Figure 10. Time-point induction of SOX2 protein in mouse colon epithelium following inactivation of *Apc* and *Sox9* genes. (**A**) IHC staining for SOX2 (top) and SOX9 (bottom) in proximal colon tissues from an A mouse (left) and an AS mouse (right) at 2, 5, 8 and 15 days following TAM injection. Scale bars, 100 μm. (**B**) IHC staining for SOX2 in proximal colon tissues from an A mouse (left) and an AS mouse (right) at 31 days following TAM injection. Both the lumen and base of the crypts are shown for the AS mice. Scale bars, 100 μm.

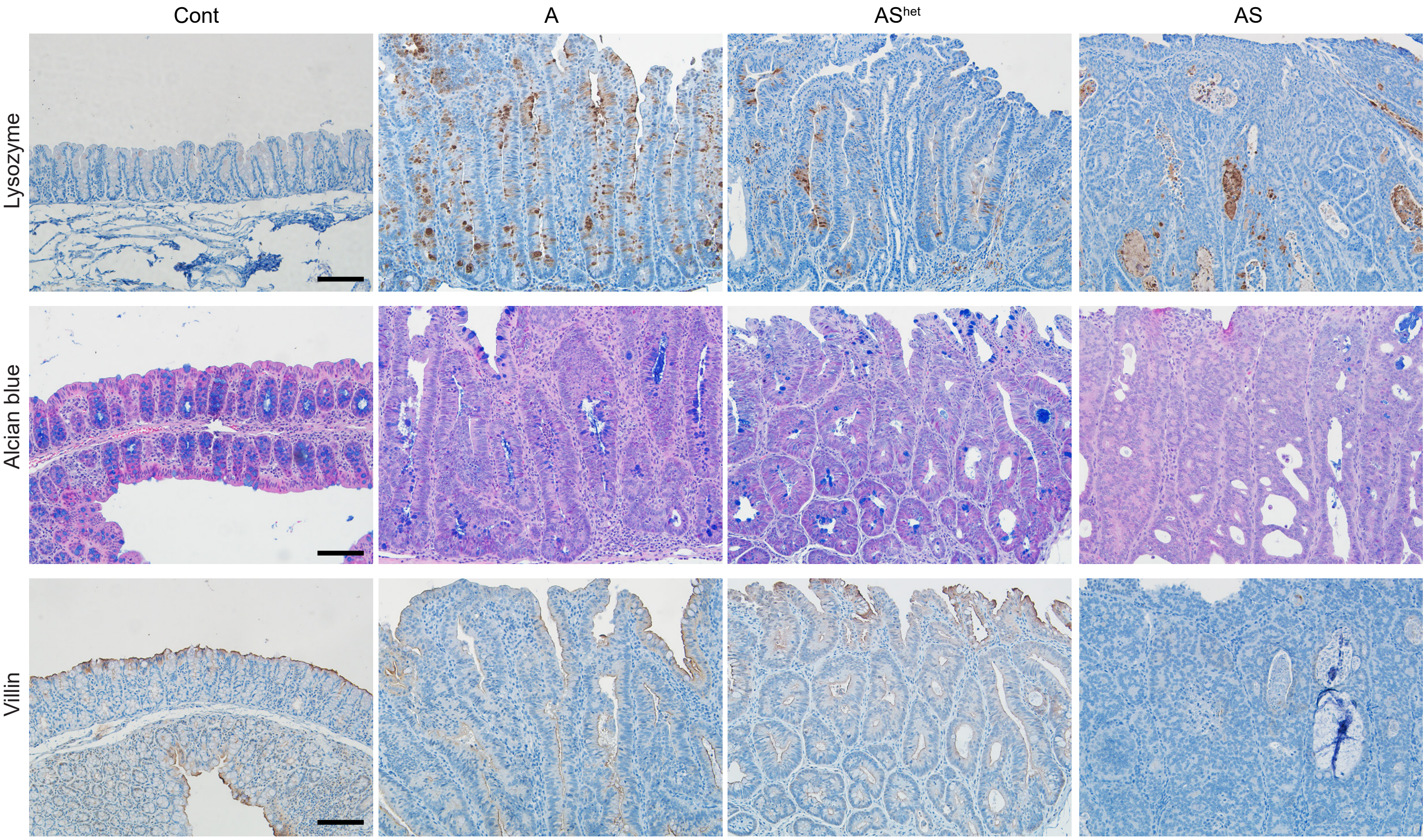

Supplemental Figure 11. Intestinal differentiation in mouse colon with inactivation of *Apc* and/or *Sox9.* IHC staining for lysozyme (Paneth cell marker; top panels) and villin (absorptive cell marker; bottom panels), and Alcian blue staining (which specifically stains goblet cells; middle panels) in proximal colon tissues from a control mouse (Cont), an A mouse, an AS^{het} mouse, and an AS mouse at 31-35 days following TAM injection. Scale bars, 100 μm.

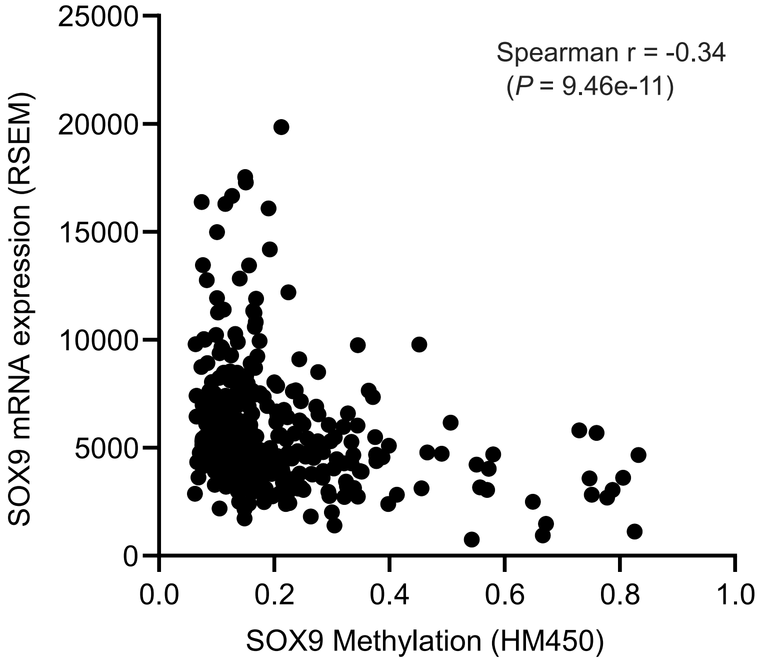

Supplemental Figure 12. Correlation plot of mRNA expression and DNA methylation levels for *SOX9* in TCGA CRC patients (*n* = 353). Spearman correlation was used to assess the relationship between *SOX9* DNA methylation and gene expression, with r = -0.34 and *P* = 9.46e-11.

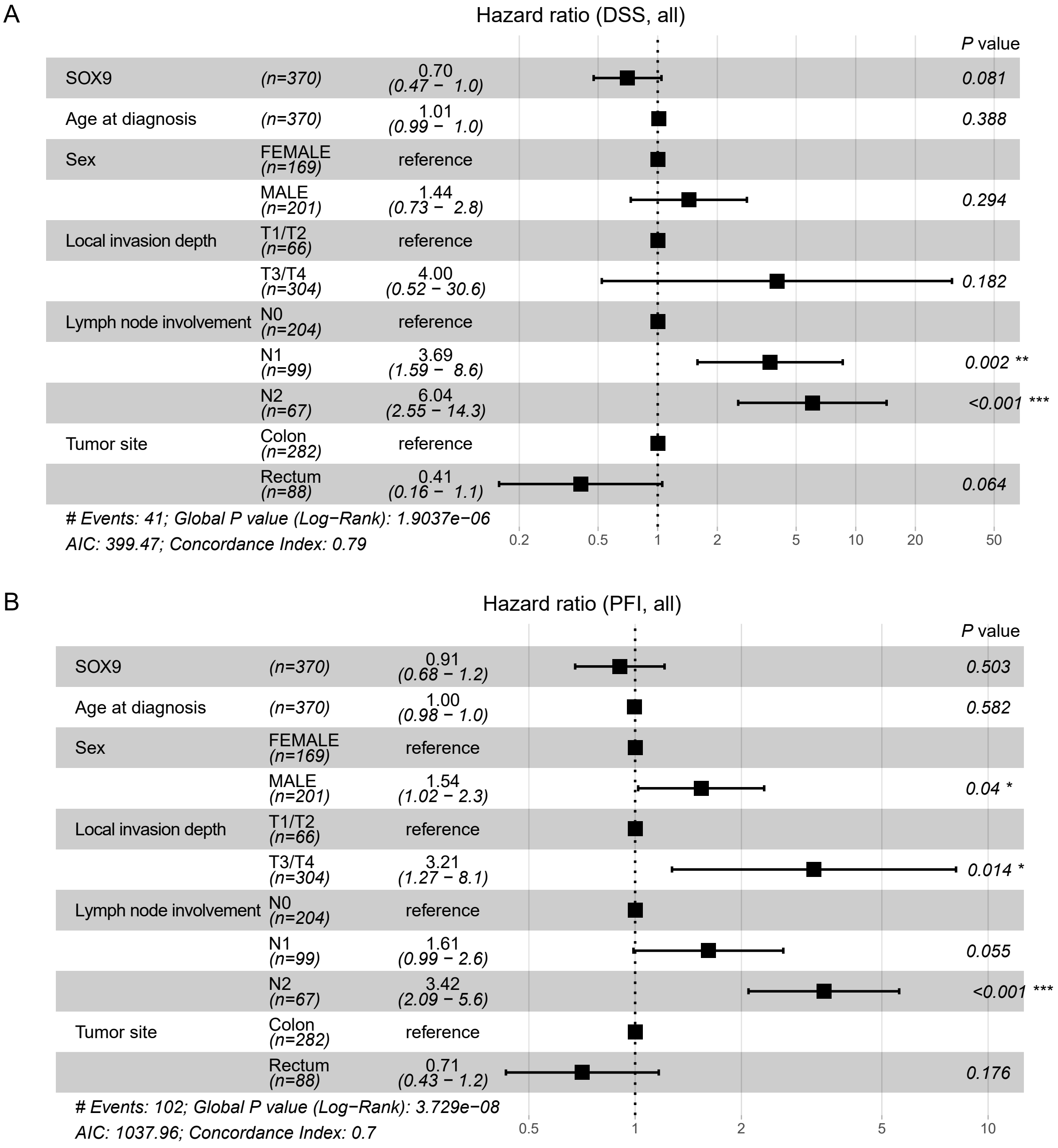

Supplemental Figure 13. Multivariate analysis of disease-specific survival (DSS), and progression-free interval (PFI) in human colorectal cancer (CRC) patients. The forest plots display the results of multivariate Cox proportional hazards models for DSS (panel **A**) and PFI (panel **B**), including *SOX9* gene expression (log_2-transformed), age at diagnosis, sex, local invasion depth, lymph node involvement, and tumor site as covariates, in CRC patients from the TCGA Colon and Rectal Cancer (COADREAD) cohort (*n* = 370, all). The square represents estimated hazard ratios, and the length of the horizontal line represents the 95% confidence interval (CI) for the hazard ratio of each covariate. *P* values for individual covariates were obtained using the Wald test (column on the far right). Statistical significance is indicated by asterisks: **P* < 0.05; ***P* < 0.01; ****P* < 0.001.

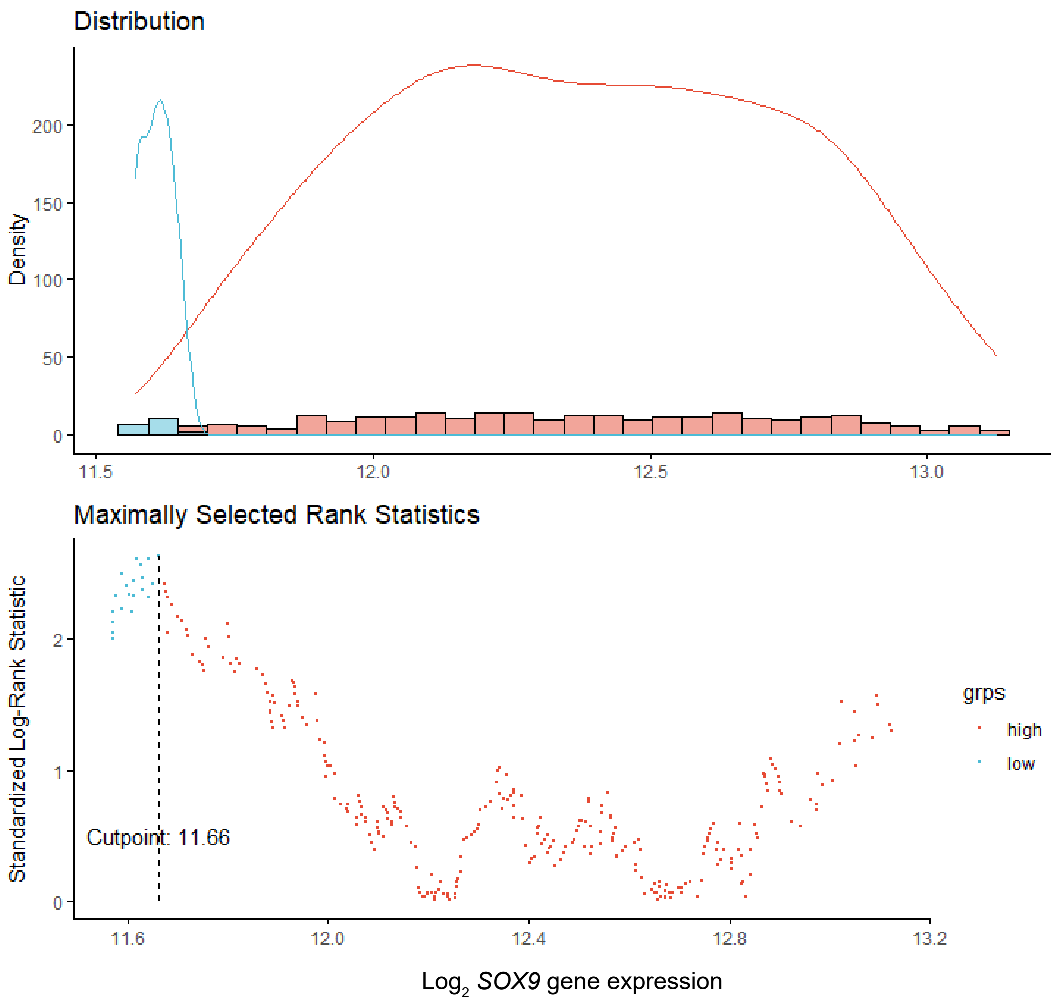

Supplemental Figure 14. Selection of the optimal cutoff value for log_2-transformed *SOX9* gene expression to divide CRC patients for survival analysis. Identification of the optimal cutoff for *SOX9* gene expression that corresponds to the most significant relation with overall survival using the R function surv_cutpoint(). The (log_2- transformed) *SOX9* gene expression value 11.66 was chosen to divide patients into *SOX9* low (<=11.66) and high (>11.66) groups, which were used for all the comparisons in this study.

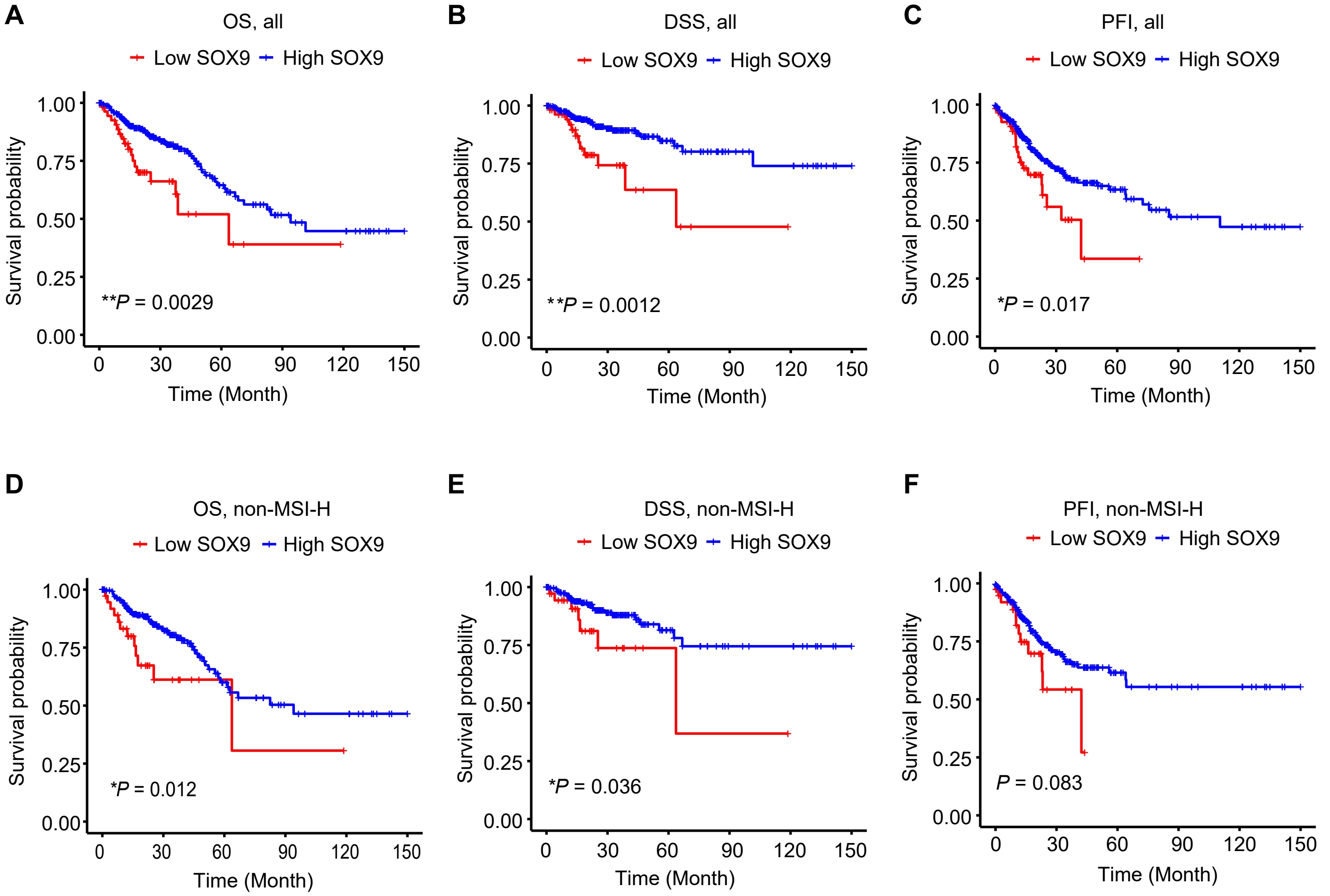

Supplemental Figure 15. Low *SOX9* gene expression is associated with decreased overall survival (OS), disease-specific survival (DSS), and progression-free interval (PFI) in patients with colorectal cancer (CRC). CRC patients from TCGA Colon and Rectal Cancer (COADREAD) cohort (n = 376) were divided into two groups based on *SOX9* gene expression (with the cutoff 11.66 chosen in Supplemental Figure 14). Kaplan–Meier survival curves illustrate OS (panels **A** and **D**), DSS (panels **B** and **E**), and PFI (panels **C** and **F**) for all CRC patients (panels **A**–**C**, n = 376) and non-MSI-H CRC patients (panels **D**–**F**, n = 288) according to SOX9 gene expression. P values were obtained by log-rank test comparing CRC patients with low and higher *SOX9* expression levels. Statistical significance is indicated by asterisks: *P < .05; **P < .01; ***P < .001.

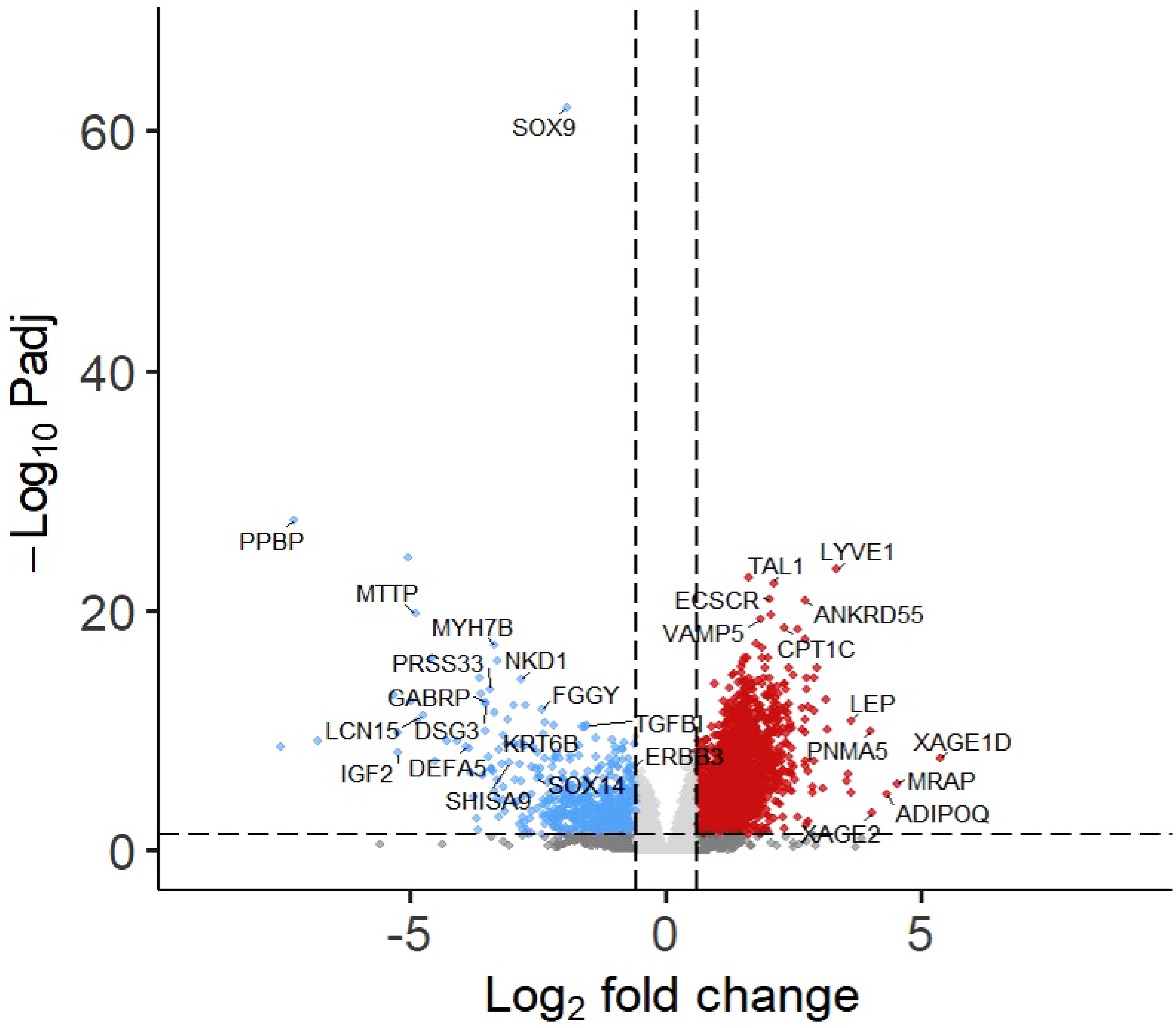

Supplemental Figure 16. Volcano plot showing differentially expressed genes (DEGs) in human CRC patients with low *SOX9* expressions versus the patients with higher *SOX9* gene expression. Significant DEGs (FDR adjusted *P* <= 0.05 and |FC| > 1.5) were highlighted in red for genes up-regulated in the *SOX9* low group (3152 genes) and in blue for those up-regulated in the *SOX9* high group (744 genes).

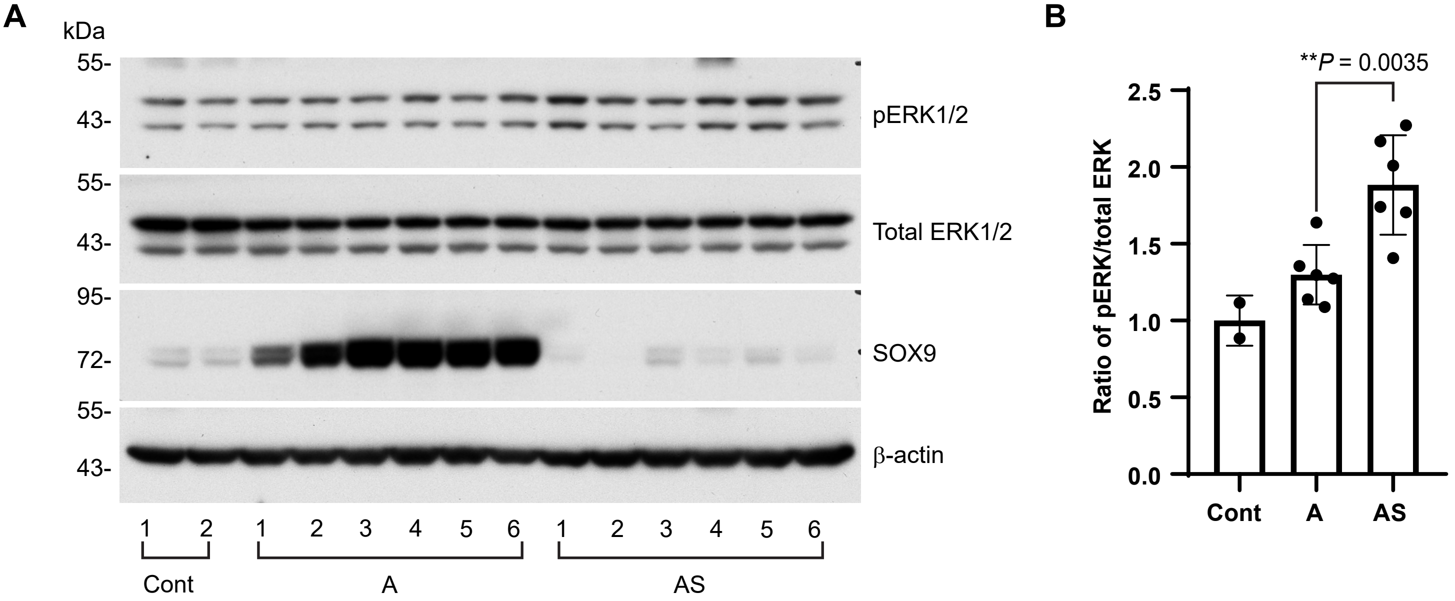

Supplemental Figure 17. Mouse colon lesions with *Apc* and *Sox9* deletion showed higher levels of phospho-ERK compared to lesions with only *Apc* deletion. (**A**) Western blot analysis of phospho-ERK1/2 (pERK1/2), total ERK1/2, SOX9, and β-actin with protein lysates from proximal colon tissues of A (n = 6), AS (n = 6), and control mice (n = 2). (**B**) Densitometry analysis of the bands for pERK1/2 and total ERK1/2 in the tissues described in panel **A**. Data are presented as a ratio of the density of pERK1/2 bands to the density of bands of the corresponding total ERK1/2 protein for each tissue sample. The average ratio for Cont samples was set as 1. $^{**}P$ = 0.0035 for the comparison of AS vs. A in Student's t test. Error bars denote SD.

Supplemental Table 1. Gene expression of selective EMT-inducing transcription factors and EMT markers in mouse and human CRCs

Mouse gene	Human gene	log2FC	FC	padj
**Mouse tissue (AS vs A)**				
Snai1	SNAI1	0.139	1.102	0.51393454
Snai2	SNAI2	0.458	1.373	0.16743503
Twist1	TWIST1	0.452	1.368	0.11976376
Epcam	EPCAM	-0.564	0.677	1.9036E-05
Cdh1	CDH1	-0.210	0.864	0.191
Cdh2	CDH2	-0.560	0.678	0.26592963
Vim	VIM	-0.744	0.597	0.05600129
**Mouse organoid (AS vs A)**				
Snai1	SNAI1	2.125	4.362	0.0003645
Snai2	SNAI2	3.557	11.772	0.1065018
Twist1	TWIST1	5.591	48.186	9.9451E-17
Epcam	EPCAM	0.139	1.101	0.11198031
Cdh1	CDH1	-0.257	0.837	0.0181
Cdh2	CDH2	3.834	14.257	1.3844E-06
Vim	VIM	2.410	5.314	0.00758346
**Human CRC (low SOX9 vs high SOX9)**				
Snai1	SNAI1	0.356	1.280	0.08987042
Snai2	SNAI2	0.774	1.710	0.00190186
Twist1	TWIST1	0.684	1.607	0.04296805
Twist2	TWIST2	1.179	2.264	7.0551E-05
Zeb1	ZEB1	1.040	2.056	1.0027E-05
Zeb2	ZEB2	1.348	2.545	2.5144E-08
Cdh1	CDH1	-0.398	0.759	0.00101767
Cdh2	CDH2	1.495	2.818	2.3725E-05
Vim	VIM	1.217	2.325	2.4005E-08

Supplemental Table 2. SOX2 expression correlates with high tumor grade, increased invasiveness, and metastasis; *Sox9* deletion enhances the rate of SOX2-positive tumors.

			SOX9		SOX2		Compared to SOX2 expression
			Positive	Negative	Positive	Negative	*P* value (Fisher's exact test)
**AKP**	**Tumor Grade**						
***n* = 10**		Low	76	0	0	76 (82.6%)	<.00001****
		High	16	0	12 (13%)	4 (4.3%)	
	**Invasive**						
		No	56	0	0	56 (60.9%)	<.00001****
		Yes	36	0	12 (13%)	24 (26.1%)	
	**Lymph node invation**		2	0	1	1	
	**Liver metastasis**		1	0	1	0	
	**Lung metastasis**		1	0	0	1	
**AKPS**	**Tumor Grade**						
***n* = 6**		Low	0	0	0	0	
		High	2	10	12 (100%)	0	
	**Invasive**						
		No	0	0	0	0	
		Yes	2	10	12 (100%)	0	
	**Lymph node invation**		3(partial +)	3	6	0	
	**Liver metastasis**		1(partial +)	3	4	0	
	**Lung metastasis**		0	1	1	0	

Statistical significance is indicated by asterisks: **P* < .05; ***P* < .01; ****P* < .001; ****P < 0.0001.

Supplemental Table 3. SOX2 expression correlates with increased metastasis in a mouse CRC model

Mouse	Lymph node invasion	Liver metastasis	Lung metastasis
**AKP**	2/11	1/11	1/11
**AKPS**	6/11	6/11	1/11

Supplemental Table 4. Gene expression of intestinal differentiation markers and stem cell markers in mouse colon tissues with inactivation of *Apc* (A) and/or *Sox9* (AS)

Gene name	log2FC	FC.easy	padj	Functions
**Differentiation markers (AS v A)**				
Lyz1	-2.54292	-5.82767	3.13E-25	Paneth Cell
Mmp7	-1.83465	-3.56685	6.31E-31	Paneth Cell
Muc2	-0.92911	-1.9041	0.001463	Secretory
Muc3a	-0.64538	-1.56415	0.005085	Secretory
Atoh1	-0.95072	-1.93283	0.009029	Secretory
Agr2	-0.79315	-1.73285	0.001562	Secretory
Fabp1	-2.66218	-6.32991	0.063327	Absorptive
B3galt5	-1.3477	-2.54506	0.00078	Absorptive
Krt20	0.249238	1.188579	0.532641	Absorptive
Vil1	-0.42879	-1.34611	0.104465	Absorptive
**Stem cell markers (AS v A)**				
Msi2	0.724681	1.652535	0.000543	stem cell
Msi1	0.210821	1.157347	0.515647	stem cell
Sox2	6.849914	115.3532	3.64E-88	stem cell
Bmi1	0.257197	1.195154	0.023613	stem cell
Smoc2	0.641671	1.560135	0.045098	stem cell
Lrig1	-0.1375	-1.1	0.385604	stem cell
Lgr5	-1.19326	-2.28668	7.94E-13	stem cell
Ascl2	-1.32361	-2.50292	2.64E-12	stem cell
Prom1	-0.03333	-1.02337	0.924503	stem cell

Supplemental Table 5. SOX9 protein expression is inversely corelated with tumor grade.

Grade	SOX9_Score		
Frequency<br>Total Percent<br>Row Percent<br>Column Percent	0(- or +/-)	1(1+ or 2+ or 3+)	Total
1	22<br>12.87<br>15.71<br>61.11	118<br>69.01<br>84.29<br>87.41	140<br>81.87
2	2<br>1.17<br>33.33<br>5.56	4<br>2.34<br>66.67<br>2.96	6<br>3.51
3	12<br>7.02<br>48.00<br>33.33	13<br>7.60<br>52.00<br>9.63	25<br>14.62
Total	36<br>21.05	135<br>78.95	171<br>100.00

Fisher's exact test, ***P* = 0.0010

Supplemental Table 6. Cancer related gene mutations in human CRCs with low versus high *SOX9* gene expression

Gene	Low *SOX9* expression (*n* = 52), N(%)	High *SOX9* expression (*n* = 302), N(%)	*P* value (Fisher's exact test)
APC	28 (48.6)	244 (79.9)	<0.0001 ***
KRAS	15 (22.9)	147 (48.3)	0.0100 *
PTEN	6 (8.6)	20 (7.2)	0.2447
SOX9	1 (2.8)	47 (14.7)	0.0041 **
TP53	32 (54.3)	204 (68.1)	0.4272
CTNNB1	9 (14.2)	17 (6.5)	0.0069 **
BRAF	7 (14.2)	41 (13.5)	1.0000
AXIN1	5 (5.7)	7 (3.1)	0.0199 *
AXIN2	5 (5.7)	15 (5.6)	0.1916
TCF7L2	7 (11.4)	32 (11.0)	0.4822
FBXW7	8 (14.3)	48 (16.0)	1.0000
NRAS	0 (0.0)	20 (6.3)	0.0545

Statistical significance is indicated by asterisks: *$P < .05$; **$P < .01$; ***$P < .001$.
