# Supplementary material for: SOX9 suppresses colon cancer via inhibiting epithelial-mesenchymal transition and SOX2 induction
Source: J Clin Invest. 2025 Apr 3;135(11):e184115. doi: 10.1172/JCI184115 (PMC12126244; doi:10.1172/JCI184115)
Supplement: Unedited blot and gel images [file jci-135-184115-s238.pdf]

Full unedited gel for Figure 6G

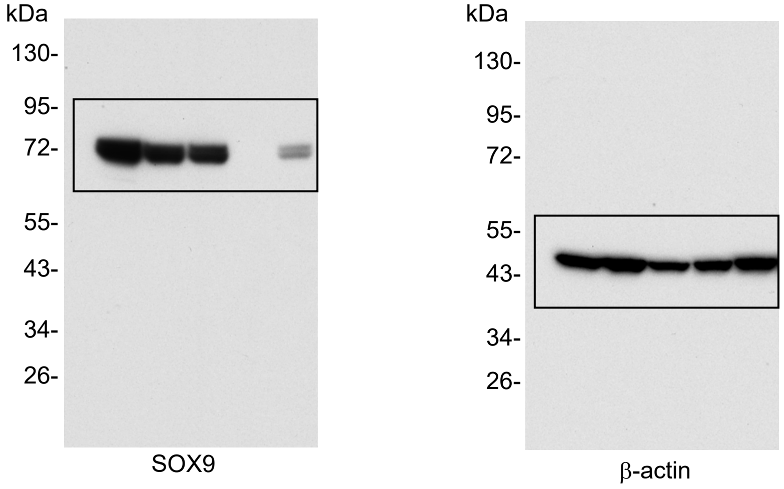

Full unedited gel for Figure 6I

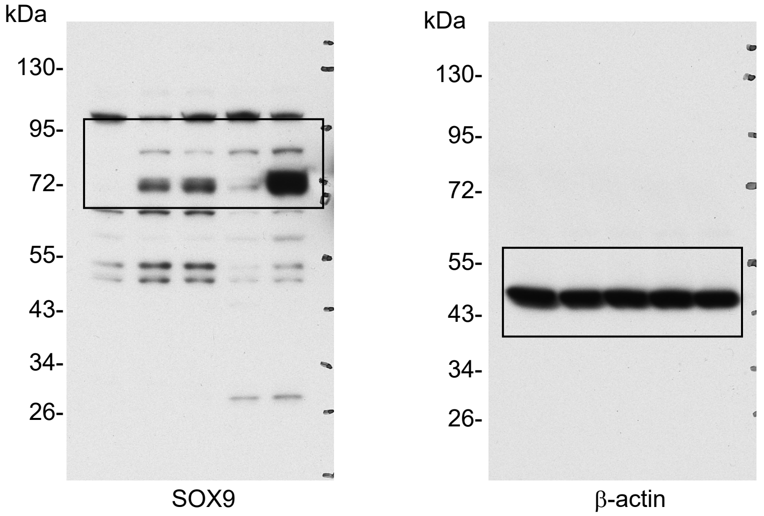

Full unedited gel for Supplemental Figure 1B

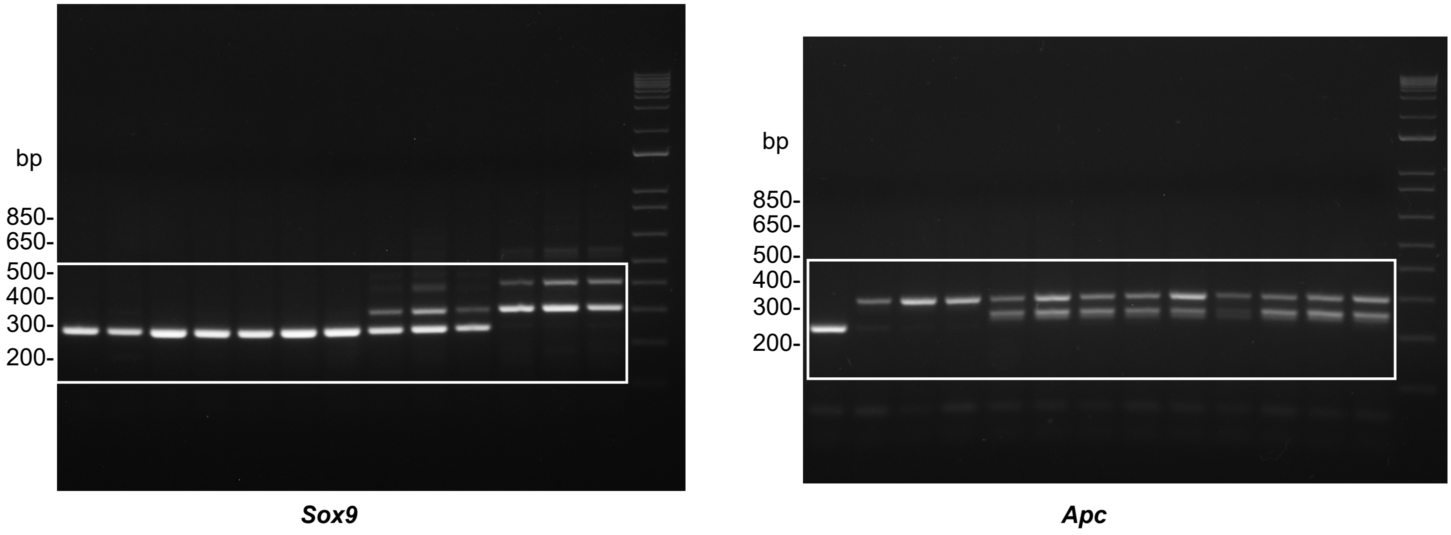

Full unedited gel for Supplemental Figure 6A

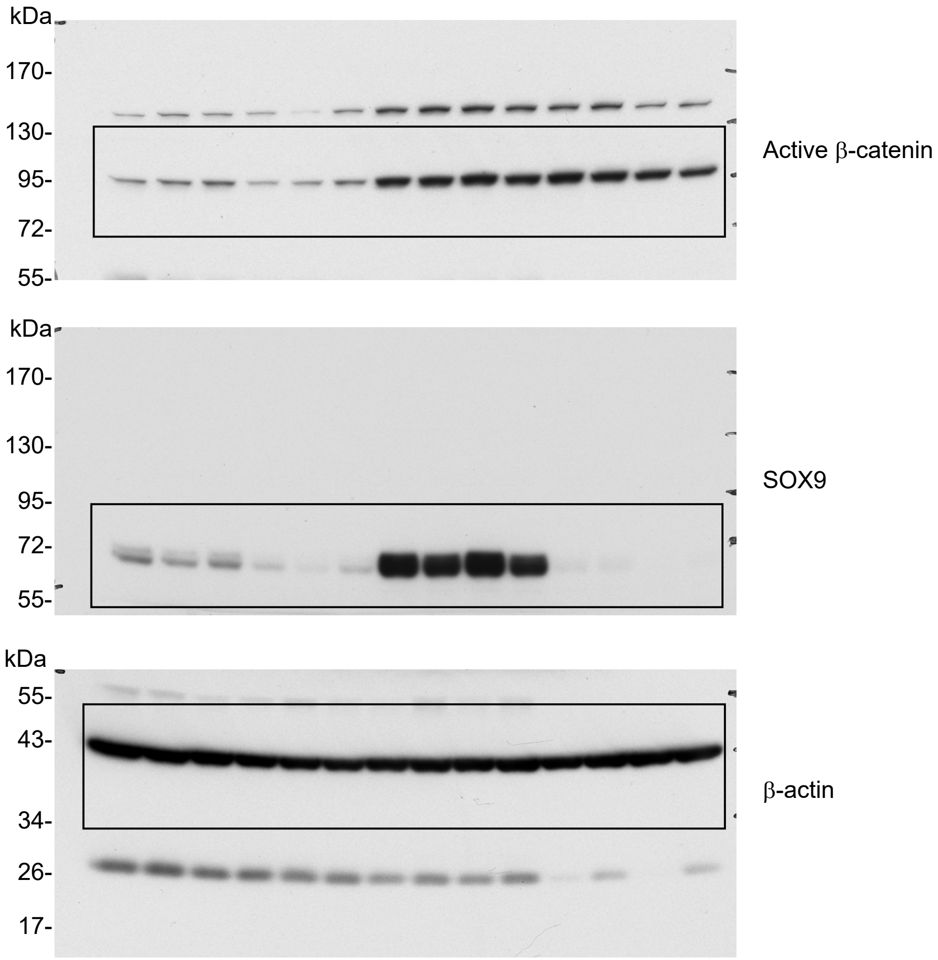

Full unedited gel for Supplemental Figure 9

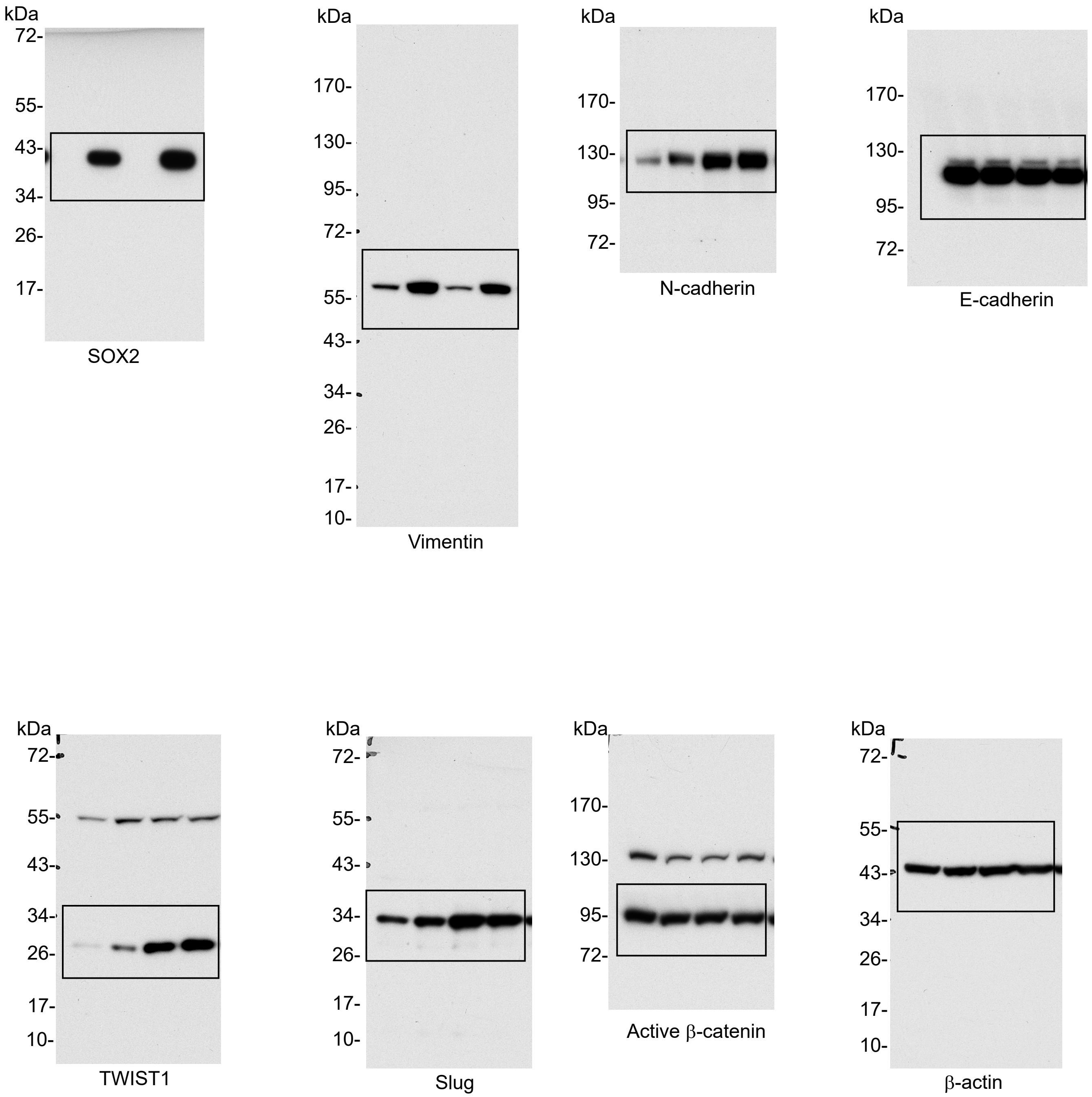

Full unedited gel for Supplemental Figure 17

kDa
55-
43-
34-

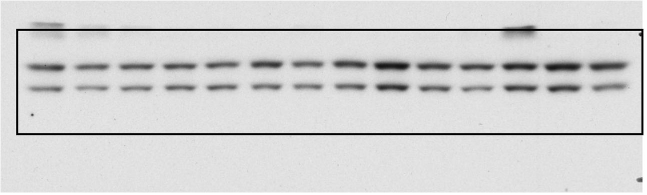

pERK1/2

kDa
55-
43-
34-

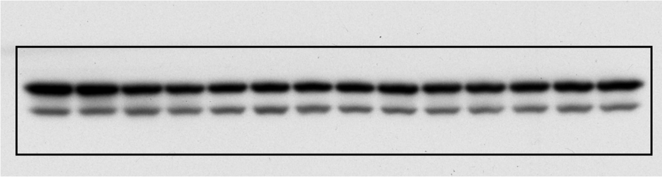

Total ERK1/2

kDa
170-
130-
95-
72-
55-

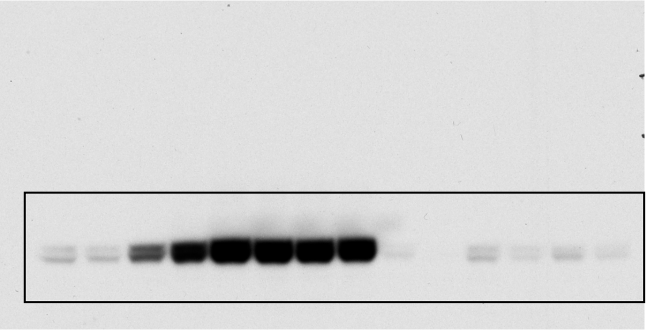

SOX9

kDa
55-
43-

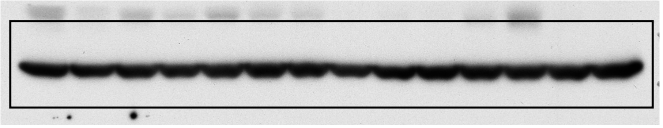

β-actin
